# Supplementary material for: Survival modelling and cost-effectiveness analysis of treatments for newly diagnosed metastatic hormone-sensitive prostate cancer
Source: PLoS One. 2022 Nov 3;17(11):e0277282. doi: 10.1371/journal.pone.0277282 (PMC9632884; doi:10.1371/journal.pone.0277282)
Supplement: S1 Appendix — (DOCX) [file pone.0277282.s001.docx]

**Survival modelling and cost-effectiveness analysis of treatments for newly diagnosed metastatic hormone-sensitive prostate cancer**

PLOS ONE

Michaela C. Barbier, Yuki Tomonaga, Dominik Menges, Henock G. Yebyo, Sarah R. Haile, Milo A. Puhan, Matthias Schwenkglenks

**Electronic S1 Appendix: Budget impact analysis**

As the cost-effectiveness analysis, the budget impact analysis was conducted as part as a Health Technology Assessment (HTA) commissioned by the Swiss Medical Board (SMB).

**Budget impact analysis methods**

The budget impact analysis consisted of two main steps. First, the total number of eligible patients per year in Switzerland was estimated. Second, the estimated number of eligible patients was combined with undiscounted cost results for the first five years after diagnosis from the cost-effectiveness analysis. Costs at the national level were estimated from the Swiss healthcare payer perspective until 2030.

## Number of eligible cases

Newly diagnosed metastatic hormone-sensitive prostate cancer (mHSPC) patients may be cases that were newly diagnosed with prostate cancer (i.e., incident cases directly staged as mHSPC) or may be already known prostate cancer cases that progressed from an earlier disease stage (prevalent cases). In Switzerland there is currently no information available on the number of patients with newly diagnosed mHSPC. However, information on the incidence and prevalence of prostate cancer is available [1,2]. To estimate the total number of mHSPC we combined national data on prostate cancer incidence and prevalence with published estimates.

### Incident cases

According to the National Institute for Cancer Epidemiology and Registration (NICER) [1], between 2012 and 2016 there were a total of 30 491 new prostate cancers diagnosed in Switzerland. Assuming that the cases were equally distributed across the years, the estimated number of cases per year was 6,098. Out of 6,068 cases, 294 cases were among males younger than 55 years (i.e., 4.82% of the total), 1,456 cases among 55–64 year-old males (23.88%), 2,588 cases among 65-74 year old males (42.44%), and 1,760 cases among males older than 75 years (28.86%).

To take into consideration the growth of the Swiss population as well as the growth of the total number of subjects at higher risk for developing prostate cancer (i.e. the ageing of the population), the estimated number of incident cases for the following years were adjusted according to the reported (from 2017 to 2019) and estimated (from 2020 to 2030) age-distribution of males in Switzerland [3,4].

To estimate the number of incident mHSPC cases, a report of the Institute for Quality and Efficiency in Health Care (IQWiG) on abiraterone treatment for prostate cancer (Bericht Nr. 605) reported that the percentage of mHSPC cases among newly diagnosed prostate cancer patients ranged from 5.6% (according to the German cancer registry) to 7.0% (according to the cancer registry of Baden-Württemberg) [5]. In the present calculation we assumed that 7% of the incident cases are directly diagnosed with mHSPC.

### Prevalent cases

For the prevalent cases we assumed that a certain percentage of already known cancer patients progresses to mHSPC. According to NICER [2], the 5-year prostate cancer prevalence in Switzerland was 28,179 cases in 2011, 26,062 cases in 2014, 25,862 cases in 2017, and 26,447 in 2020 (S1 Appendix S1 Table). For the years between 2011-2014, 2014-2017, and 2017-2020 we assumed linear decreases/increases.

**S1 Table. Five-year prostate cancer prevalence in Switzerland.**

|  | 0-49 years | 50-59 years | 60-69 years | 70-79 years | >80 years | Total |
| --- | --- | --- | --- | --- | --- | --- |
| 2011 | 147 | 2,480 | 10,504 | 10,584 | 4,465 | 28,179 |
| 2014 | 148 | 2,247 | 9,417 | 10,227 | 4,024 | 26,062 |
| 2017 | 160 | 2,167 | 9,169 | 10,198 | 4,168 | 25,862 |
| 2020 | 156 | 2,149 | 9,176 | 10,603 | 4,363 | 26,447 |

To estimate the fraction of mHSPC patients among the total population of prevalent prostate cancer patients we used estimates published in 2015 [6]. Scher et al. developed a dynamic transition model to estimate the prevalence of prostate cancer clinical states between 2009 and 2020 in the US. According to data provided in their supplementary documents, the percentage of prostate cancer patients with newly diagnosed, metastatic disease ranged from 1.6% in 2009 to 1.4% in 2020 (NB: from 2012 to 2020 the percentage remained stable at around 1.4%). We assumed that the reported percentages reflect the proportion of prevalent prostate cancer patients that are newly diagnosed as metastatic in a given year. For the years 2012 to 2020, we used the percentage as reported in the publication, whereas from 2021 onwards we applied the percentage estimate reported for 2020. These percentages were multiplied with the estimated number of prevalent cases reported by NICER (using the 5-year prevalence) [1,2]. As for the incident cases, to take into consideration the general increase and ageing of the Swiss population, the estimations for the following years (2021-2023) were adjusted according to the projected age-distribution among males in Switzerland [3,4].

## Inputs for cost and market shares

For each eligible case the undiscounted costs accrued during the first 5 years after diagnosis were included. The costs estimates were based on the results of the cost-effectiveness analysis and are summarized in S1 Appendix S2 Table. The costs included drug and administration costs, adverse effects costs, imaging costs, palliative care, and end of life costs.

**S2 Table. Estimated mean yearly costs per patient by treatment strategy (EUR, undiscounted).**

|  | Year 1 | Year 2 | Year 3 | Year 4 | Year 5 | Total |
| --- | --- | --- | --- | --- | --- | --- |
| ADT alone | 6,953 | 7,109 | 4,431 | 3,167 | 2,323 | 23,982 |
| ADT+apalutamide | 36,229 | 28,388 | 22,957 | 19,522 | 15,756 | 122,854 |
| ADT+enzalutamide | 35,758 | 28,233 | 23,076 | 19,802 | 16,099 | 122,968 |
| ADT+docetaxel | 14,742 | 6,155 | 3,919 | 2,922 | 2,233 | 29,972 |
| ADT+abiraterone | 16,520 | 13,753 | 10,805 | 9,038 | 7,375 | 57,490 |

ADT, androgen deprivation therapy; EUR, euros.

With the currently available information it was difficult to define a standard of care scenario. There is no published information on the treatment distribution among mHSPC patients (i.e., which percentage receives androgen deprivation therapy (ADT) alone, ADT + docetaxel, etc.). According to expert opinion, there is a high variability in the use of the investigated treatments across hospitals/regions, patient characteristics (age, physical status, comorbidities), cancer burden (high vs. low volume), and treating physicians (urologists vs. oncologists). Moreover, while ADT and docetaxel have been introduced and used in Switzerland since many years, the other treatments entered the Swiss market with the indication for mHSPC only recently. It is unknown whether there were patients that switched from a treatment with ADT alone to a combination treatment (e.g., ADT + abiraterone).

In light of this, we decided for a standard of care treatment assumption that may represent the clinical practice before the marketing approval of abiraterone, enzalutamide, and apalutamide: in this scenario we assumed that 50% of the patients would receive ADT alone, whereas 50% would receive ADT + docetaxel. Alternative assumptions using other marked shares are reported in S1 Appendix S3 Table.

**S3 Table. Treatment distributions for standard of care and alternatives assumptions.**

| Assumptions | ADT alone | ADT + docetaxel | ADT + abiraterone | ADT + enzalutamide | ADT + apalutamide |
| --- | --- | --- | --- | --- | --- |
| Standard of care | 50% | 50% | - | - | - |
| Assumption 1 | 50% | 25% | 25% | - | - |
| Assumption 2 | 40% | 40% | 20% | - | - |
| Assumption 3 | 40% | 20% | 20% | 20% | - |
| Assumption 4 | 20% | 20% | 20% | 20% | 20% |
| Assumption 5 | - | 50% | 50% | - | - |
| Assumption 6 | 100% | - | - | - | - |
| Assumption 7 | - | 100% | - | - | - |
| Assumption 8 | - | - | 100% | - | - |
| Assumption 9 | - | - | - | 100% | - |
| Assumption 10 | - | - | - | - | 100% |

ADT, androgen deprivation therapy.

## Sensitivity analysis assumptions

In the sensitivity analysis we first varied the assumptions concerning the percentages of incident and prevalent prostate cancer having mHSPC by ±30%. For the incidence, the estimated percentage of cancer patients being directly diagnosed with mHSPC was varied from 5% to 9% (this range included the variation in the estimations identified in the published literature). For the prevalence, the estimated percentage of prevalent cases that are newly diagnosed with mHSPC per year was varied from 0.94% to 1.86%. Second, we varied the estimated mean yearly costs per patient by ±30%. Third, to consider that incident and prevalent cases might be double counted (if for example incident cases are also counted as prevalent from the beginning), a sensitivity analysis assuming up to 50% overlap of incident and prevalent cases was conducted.

**Budget impact analyses results**

## Number of eligible cases

S1 Appendix S4 Table summarises the estimated number of newly diagnosed mHSPC cases depending on prostate cancer incidence and prevalence. The estimated total number of cases increased from 796 in 2018 to 1,031 in 2030 (+ 30%).

## Budget impact

S1 Appendix S5 Table illustrates the total costs according to the assumed marked shares, while S1 Appendix S6 Table shows the difference in total costs per year, relative to the standard of care assumption (50% ADT alone and 50% ADT + docetaxel).

The estimated total costs of mHSPC management in Switzerland strongly depended on the treatment strategy. Based on the hypothetical assumption that all mHSPC patients would be treated with the same drugs (assumptions 6-10), the estimated total costs in 2021 were estimated at EUR 19.8 million for ADT alone, EUR 25.0 million for ADT + docetaxel, EUR 47.4 million for ADT + abiraterone, EUR 101.3 million for ADT + apalutamide, , and EUR 101.3 million for ADT + enzalutamide. Alternative assumptions would simply be linear combinations of these costs and lead to total budgets ranging from EUR 27.4 million to EUR 59.0 million in 2021. If compared to our main standard of care assumption (50% ADT alone + 50% ADT + docetaxel), a combination of 50% ADT alone, 25% ADT + docetaxel, 25% ADT + abiraterone (assumption 1) would lead to a budget impact increase of EUR 5.6 million in 2021 (i.e., from EUR 22.4 million to EUR 28.0 million).

## Sensitivity analysis

In the first sensitivity analysis the numbers of incident and prevalent prostate cancer cases were varied. With 30% lower incidence and prevalence of mHSPC, the estimated number of eligible cases was 600 in 2021 and 722 in 2030. If a 30% higher incidence and prevalence was assumed, the estimated number of cases was 1,114 in 2021 and 1,340 in 2030. Since the budget impact was calculated by multiplying the number of cases with the estimated costs per case, a 30% change in the costs led to the same results as a 30% change in the incidence/prevalence of mHSPC.

In the sensitivity analysis assuming that incident and prevalent cases were partially overlapping, the estimated number of eligible cases ranged from 611 (50% overlap) to 807 (10% overlap) in 2021 and from 735 (50% overlap) to 972 (10% overlap) in 2030. For the standard of care assumption, this led to total costs ranging from EUR 16.0 million (50% overlap) to EUR 22.4 million (10% overlap) in 2021 and from EUR 19.3 million (50% overlap) to EUR 27.1 million (10% overlap) in 2030.

**S4 Table. Estimated number of mHSPC cases depending on prostate cancer incidence and prevalence.**

| **Incidence of prostate cancer** | | | | | | | | | | | | | |
| --- | --- | --- | --- | --- | --- | --- | --- | --- | --- | --- | --- | --- | --- |
| Year | **2018** | **2019** | **2020** | **2021** | **2022** | **2023** | **2024** | **2025** | **2026** | **2027** | **2028** | **2029** | **2030** |
| Total | 6,360 | 6,501 | 6,861 | 7,021 | 7,178 | 7,336 | 7,494 | 7,653 | 7,811 | 7,971 | 8,130 | 8,292 | 8,444 |
| **Estimated number of newly diagnosed mHSPC (among incident cases)** | | | | | | | | | | | | | |
| % of total | 7% | 7% | 7% | 7% | 7% | 7% | 7% | 7% | 7% | 7% | 7% | 7% | 7% |
| N | 445 | 455 | 480 | 491 | 502 | 514 | 525 | 536 | 547 | 558 | 569 | 580 | 591 |
| **5-year prevalence of prostate cancer** | | | | | | | | | | | | | |
| Year | **2018** | **2019** | **2020** | **2021** | **2022** | **2023** | **2024** | **2025** | **2026** | **2027** | **2028** | **2029** | **2030** |
| Total | 26,057 | 26,252 | 26,447 | 27,033 | 27,639 | 28,260 | 28,913 | 29,551 | 30,182 | 30,795 | 31,395 | 31,986 | 32,562 |
| **Estimated number of newly diagnosed mHSPC (among prevalent cases)** | | | | | | | | | | | | | |
| % of total | 1.344% | 1.344% | 1.351% | 1.351% | 1.351% | 1.351% | 1.351% | 1.351% | 1.351% | 1.351% | 1.351% | 1.351% | 1.351% |
| N | 350 | 353 | 357 | 365 | 373 | 382 | 390 | 399 | 408 | 416 | 424 | 432 | 440 |
| **Estimated total number of newly diagnosed mHSPC patients** | | | | | | | | | | | | | |
| Year | **2018** | **2019** | **2020** | **2021** | **2022** | **2023** | **2024** | **2025** | **2026** | **2027** | **2028** | **2029** | **2030** |
| Total | 796 | 808 | 837 | 857 | 876 | 895 | 915 | 935 | 954 | 974 | 993 | 1,012 | 1,031 |

mHSPC, metastatic hormone-sensitive prostate cancer.

**S5 Table. Estimated total costs per year (in million EUR, undiscounted).**

| Total costs per year (million EUR) | | | | | | | | | | | | | |
| --- | --- | --- | --- | --- | --- | --- | --- | --- | --- | --- | --- | --- | --- |
|  | 2018 | 2019 | 2020 | 2021 | 2022 | 2023 | 2024 | 2025 | 2026 | 2027 | 2028 | 2029 | 2030 |
| Standard of care | 21.3 | 21.5 | 21.9 | 22.4 | 22.9 | 23.5 | 24.0 | 24.6 | 25.1 | 25.6 | 26.1 | 26.5 | 27.1 |
| Assumption 1 | 26.7 | 26.9 | 27.4 | 28.0 | 28.7 | 29.3 | 30.0 | 30.7 | 31.4 | 32.0 | 32.7 | 33.1 | 33.8 |
| Assumption 2 | 26.1 | 26.3 | 26.8 | 27.4 | 28.1 | 28.7 | 29.4 | 30.0 | 30.7 | 31.3 | 32.0 | 32.4 | 33.1 |
| Assumption 3 | 40.7 | 41.0 | 41.8 | 42.7 | 43.7 | 44.7 | 45.8 | 46.8 | 47.8 | 48.8 | 49.8 | 50.5 | 51.6 |
| Assumption 4 | 56.3 | 56.7 | 57.7 | 59.0 | 60.3 | 61.7 | 63.2 | 64.6 | 66.0 | 67.4 | 68.8 | 69.8 | 71.2 |
| Assumption 5 | 34.5 | 34.8 | 35.4 | 36.2 | 37.0 | 37.9 | 38.8 | 39.7 | 40.5 | 41.4 | 42.2 | 42.8 | 43.7 |
| Assumption 6 | 18.9 | 19.0 | 19.4 | 19.8 | 20.3 | 20.8 | 21.3 | 21.7 | 22.2 | 22.7 | 23.1 | 23.5 | 23.9 |
| Assumption 7 | 23.7 | 23.9 | 24.5 | 25.0 | 25.6 | 26.2 | 26.8 | 27.4 | 28.0 | 28.6 | 29.1 | 29.4 | 30.2 |
| Assumption 8 | 45.3 | 45.6 | 46.4 | 47.4 | 48.5 | 49.6 | 50.8 | 51.9 | 53.1 | 54.2 | 55.3 | 56.1 | 57.3 |
| Assumption 9 | 96.8 | 97.6 | 99.2 | 101.3 | 103.6 | 106.1 | 108.6 | 111.1 | 113.5 | 115.9 | 118.3 | 120.0 | 122.5 |
| Assumption 10 | 96.7 | 97.5 | 99.2 | 101.3 | 103.6 | 106.0 | 108.6 | 111.0 | 113.4 | 115.8 | 118.2 | 119.9 | 122.4 |
| Note: Since most of the investigated treatments were introduced in the Swiss marked only recently, cost estimates are only reported starting from 2018.  ADT, androgendeprivation therapy; EUR, Euros; mHSPC, metastatic hormone-sensitive prostate cancer.  Assumption description:  Standard of care: 50% ADT alone, 50% ADT + docetaxel  Assumption 1: 50% ADT alone, 25% ADT + docetaxel, 25% ADT + abiraterone  Assumption 2: 40% ADT alone, 40% ADT + docetaxel, 20% ADT + abiraterone  Assumption 3: 40% ADT alone, 20% ADT + docetaxel, 20% ADT + abiraterone, 20% ADT + enzalutamide  Assumption 4: 20% ADT, 20% ADT + docetaxel, 20% ADT + abiraterone, 20% ADT + enzalutamide, 20% ADT + apalutamide  Assumption 5: 50% ADT + docetaxel, 50% ADT + abiraterone  Assumption 6: 100% ADT alone  Assumption 7: 100% ADT + docetaxel  Assumption 8: 100% ADT + abiraterone  Assumption 9: 100% ADT + enzalutamide  Assumption 10: 100% ADT + apalutamide | | | | | | | | | | | | | |

**S6 Table Total costs difference per year in comparison to standard of care (in million EUR, undiscounted).**

| Total costs per year (million EUR) | | | | | | | | | | | | | |
| --- | --- | --- | --- | --- | --- | --- | --- | --- | --- | --- | --- | --- | --- |
|  | 2018 | 2019 | 2020 | 2021 | 2022 | 2023 | 2024 | 2025 | 2026 | 2027 | 2028 | 2029 | 2030 |
| Standard of care | - | - | - | - | - | - | - | - | - | - | - | - | - |
| Assumption 1 | 5.4 | 5.4 | 5.5 | 5.6 | 5.7 | 5.9 | 6.0 | 6.1 | 6.3 | 6.4 | 6.5 | 6.7 | 6.8 |
| Assumption 2 | 4.8 | 4.8 | 4.9 | 5.0 | 5.1 | 5.2 | 5.4 | 5.5 | 5.6 | 5.7 | 5.8 | 5.9 | 6.0 |
| Assumption 3 | 19.4 | 19.6 | 19.8 | 20.2 | 20.7 | 21.2 | 21.7 | 22.2 | 22.7 | 23.2 | 23.7 | 24.0 | 24.5 |
| Assumption 4 | 35.0 | 35.2 | 35.8 | 36.5 | 37.4 | 38.3 | 39.2 | 40.1 | 40.9 | 41.8 | 42.7 | 43.3 | 44.2 |
| Assumption 5 | 13.2 | 13.3 | 13.5 | 13.8 | 14.1 | 14.4 | 14.8 | 15.1 | 15.4 | 15.8 | 16.1 | 16.3 | 16.7 |
| Assumption 6 | -2.4 | -2.4 | -2.5 | -2.6 | -2.7 | -2.7 | -2.8 | -2.8 | -2.9 | -2.9 | -3.0 | -3.0 | -3.1 |
| Assumption 7 | 2.4 | 2.4 | 2.5 | 2.6 | 2.7 | 2.7 | 2.8 | 2.8 | 2.9 | 2.9 | 3.0 | 3.0 | 3.1 |
| Assumption 8 | 24.0 | 24.1 | 24.5 | 24.9 | 25.5 | 26.1 | 26.8 | 27.4 | 28.0 | 28.6 | 29.2 | 29.6 | 30.2 |
| Assumption 9 | 75.5 | 76.1 | 77.3 | 78.9 | 80.7 | 82.6 | 84.6 | 86.5 | 88.4 | 90.3 | 92.1 | 93.5 | 95.4 |
| Assumption 10 | 75.4 | 76.0 | 77.2 | 78.8 | 80.6 | 82.6 | 84.6 | 86.4 | 88.3 | 90.2 | 92.1 | 93.4 | 95.3 |
| Note: Since most of the investigated treatments were introduced in the Swiss marked only recently, cost estimates are only reported starting from 2018.  ADT, androgendeprivation therapy; EUR, Euros; mHSPC, metastatic hormone-sensitive prostate cancer.  Assumption description:  Standard of care: 50% ADT alone, 50% ADT + docetaxel.  Assumption 1: 50% ADT alone, 25% ADT + docetaxel, 25% ADT + abiraterone.  Assumption 2: 40% ADT alone, 40% ADT + docetaxel, 20% ADT + abiraterone.  Assumption 3: 40% ADT alone, 20% ADT + docetaxel, 20% ADT + abiraterone, 20% ADT + enzalutamide.  Assumption 4: 20% ADT, 20% ADT + docetaxel, 20% ADT + abiraterone, 20% ADT + enzalutamide, 20% ADT + apalutamide.  Assumption 5: 50% ADT + docetaxel, 50% ADT + abiraterone.  Assumption 6: 100% ADT alone.  Assumption 7: 100% ADT + docetaxel.  Assumption 8: 100% ADT + abiraterone.  Assumption 9: 100% ADT + enzalutamide.  Assumption 10: 100% ADT + apalutamide. | | | | | | | | | | | | | |

**References**

1. National Institute for Cancer Epidemiology and Registration (NICER). National statistics on cancer incidence - whole of Switzerland and language regions. [cited 17 Oct 2022]. Available: https://www.nicer.org/en/statistics-atlas/cancer-incidence/

2. National Institute for Cancer Epidemiology and Registration (NICER). Cancer prevalence in Switzerland 2005 - 2020: estimated number of prevalent subjects. [cited 17 Oct 2022]. Available: https://www.nicer.org/en/statistics-atlas/cancer-prevalence/

3. Swiss Federal Statistical Office. Altersmasszahlen der ständigen Wohnbevölkerung nach Staatsangehörigkeitskategorie und Geschlecht, 1999-2019. [cited 17 Oct 2022]. Available: https://www.bfs.admin.ch/bfs/de/home/statistiken/bevoelkerung/alterung.assetdetail.14367971.html

4. Swiss Federal Statistical Office. Altersmasszahlen der ständigen Wohnbevölkerung nach Staatsangehörigkeitskategorie und Geschlecht, Schätzungen für 2020-2030. [cited 17 Oct 2022]. Available: https://www.pxweb.bfs.admin.ch/pxweb/de/

5. IQWiG-Berichte - Nr. 605 - Abirateroneacetat (Prostatakarzinom). 2018 [cited 17 Oct 2022]. Available: https://www.iqwig.de/de/projekte-ergebnisse/projekte/arzneimittelbewertung/2017/a17-64-abirateronacetat-prostatakarzinom-nutzenbewertung-gemaess-35a-sgb-v.8672.html

6. Scher HI, Solo K, Valant J, Todd MB, Mehra M. Prevalence of Prostate Cancer Clinical States and Mortality in the United States: Estimates Using a Dynamic Progression Model. PloS one. 2015;10: e0139440. doi:10.1371/journal.pone.0139440
