# Supplementary material for: Survival modelling and cost-effectiveness analysis of treatments for newly diagnosed metastatic hormone-sensitive prostate cancer
Source: PLoS One. 2022 Nov 3;17(11):e0277282. doi: 10.1371/journal.pone.0277282 (PMC9632884; doi:10.1371/journal.pone.0277282)
Supplement: S2 Appendix — (DOCX) [file pone.0277282.s002.docx]

**Survival modelling and cost-effectiveness analysis of treatments for newly diagnosed metastatic hormone-sensitive prostate cancer**

PLOS ONE

Michaela C. Barbier, Yuki Tomonaga, Dominik Menges, Henock G. Yebyo, Sarah R. Haile, Milo A. Puhan, Matthias Schwenkglenks

**Corresponding Author:**

Dr. Michaela Carla Barbier, Institute of Pharmaceutical Medicine, University of Basel, Klingelbergstrasse 61, CH-4056 Basel, Switzerland. E-mail: [michaela.barbier@unibas.ch](mailto:michaela.barbier@unibas.ch)

**Electronic S2 Appendix: Cost-effectiveness analysis**

Contents

[S1 Text. Progression-free survival definition. 4](#_Toc117106741)

[S2 Text. Study eligibility. 4](#_Toc117106742)

[S3 Text. Modelling Survival and Progression of metastatic disease. 5](#_Toc117106743)

[S4 Text. Treatment strategies. 5](#_Toc117106744)

[S5 Text. Adverse effects. 6](#_Toc117106745)

[S6 Text. Further line treatments. 7](#_Toc117106746)

[S7 Text. End of life hospitalization costs. 7](#_Toc117106747)

[S8 Text. Uncertainty analyses. 8](#_Toc117106748)

[S9 Text. Survival analysis results. 8](#_Toc117106749)

[S1 Table. Studies considered for estimation of survival curves. 9](#_Toc117106750)

[S2 Table. Adverse effect monthly rates for ADT and monthly incidence rate ratios for combination therapies. 10](#_Toc117106751)

[S3 Table. Post-progression treatments. 10](#_Toc117106752)

[S4 Table. Proportion of patients with CRPC 1L, CRPC 2L, and late-stage palliative care treatment. 11](#_Toc117106753)

[S5 Table. CRPC first-line and CRPC second-line treatment durations. 11](#_Toc117106754)

[S6 Table. Routine care and monitoring resource use. 12](#_Toc117106755)

[S7 Table. Imaging resource use. 12](#_Toc117106756)

[S8 Table. Drug and drug administration costs for mHSPC treatment. 13](#_Toc117106757)

[S9 Table. Further mHSPC cost inputs. 14](#_Toc117106758)

[S10 Table. Utility input parameters. 15](#_Toc117106759)

[S11 Table. Scenario analyses. 16](#_Toc117106760)

[S12 Table. Median survival results of recreated and pooled IPD. 17](#_Toc117106761)

[S13 Table. Hazard ratios of base case and scenario analyses. 18](#_Toc117106762)

[S14 Table. Base case lifetime cost results per cost component for each treatment strategy (discounted, in EUR). 19](#_Toc117106763)

[S15 Table. Main scenario analysis results. 20](#_Toc117106764)

[S16 Table.Scenario analysis results for apalutamide price reduction. 22](#_Toc117106765)

[S17 Table. Scenario analysis results for enzalutamide price reduction. 23](#_Toc117106766)

[S1 Fig. Markov model bubble diagram for the treatment of mHSPC. 24](#_Toc117106767)

[S2 Fig. Pooled PFS curve for ADT and re-created Kaplan Meier PFS estimates for the ADT arms of each trial. 25](#_Toc117106768)

[S3 Fig. Pooled and extrapolated OS and PFS curves for ADT with re-created individual patient survival data. 26](#_Toc117106769)

[S4 Fig. Estimated PFS curves for ADT and intervention strategies (gamma extrapolation). 27](#_Toc117106770)

[S5 Fig. PFS PH assumption results (Schoenfeld test) and Kaplan-Meier curves from re-created individual patient data: (a) GETUG/Gravis 2013 (b) CHAARTED/Sweeney 2015 (c) STAMPEDE/Clarke 2019 (d) TITAN/Chi 2019 (e) ARCHES/Armstrong 2019. 28](#_Toc117106771)

[S6 Fig. Tornado graph base case (ADT+docetaxel versus ADT+abiraterone). 33](#_Toc117106772)

[S7 Fig. Tornado graph base case (ADT+docetaxel versus ADT). 34](#_Toc117106773)

[S8 Fig. Cost-effectiveness acceptability curve 35](#_Toc117106774)

[References 36](#_Toc117106775)

# S1 Text. Progression-free survival definition.

We considered the following outcomes as deemed most closely reflecting a meaningful clinical progression in the following order of priority:

- Clinical progression-free survival (cPFS): time to progression in clinical symptoms or radiographic findings, or death or
- Radiographic progression-free survival (rPFS): time to progression in radiographic findings, or death.

We did not consider the following definitions of progression-free survival (PFS) in the cost-effectiveness analysis:

- Failure-free survival (FFS): Defined as the time from randomization to first clinical, radiographic or biochemical (prostate specific antigen (PSA)) progression, or death.
- Biochemical (PSA) progression-free survival (bPFS): Defined as the time from randomization to first biochemical (PSA) progression, or death.

# S2 Text. Study eligibility.

LATITUDE was excluded in the base case analysis as it only included high-risk patients. The findings of this trial were therefore not generalizable to our overall patient population.

Since the overall STAMPEDE trial included a substantial proportion of high-risk, non-metastatic prostate cancer patients in addition to metastatic patients (61% in the docetaxel part of the trial; 52% in the abiraterone part of the trial) [1,2], we only included data of patients with metastatic disease from both parts. In addition, for the abiraterone part, we used overall survival (OS), but no PFS data, since available FFS outcomes did not match our PFS endpoint definitions of either cPFS or rPFS.

The ENZAMET trial evaluated androgen deprivation therapy (ADT) plus enzalutamide versus ADT plus non-steroidal anti-androgen [3]. The main reason for excluding the study from the base case was that early administration of docetaxel was planned in 45% of the patients (61% with high-volume disease, 27% with low-volume). We were only interested in the effect of enzalutamide without docetaxel. Subgroup results from ENZAMET were available but represented a high proportion of patients with low-volume disease, potentially leading to baseline risks for progression and death different for those to be expected in an average metastatic hormone-sensitive prostate cancer (mHSPC) patient population.

NCT02058706 [4] also compared ADT+enzalutamide to ADT. We did not include the trial in our analysis either because NCT02058706 is only a phase II trial and had the aim to enrich the trial population with black individuals. In addition, bicalutamide was administered continuously and not only during the first 30 days in combination with ADT (the latter also holds for ENZAMET).

With regard to the TITAN trial comparing ADT+apalutamide to ADT, we used the final OS data from the Chi et al. 2021 publication [5]. Due to fact that only bPFS and not rPFS data were updated in the final analysis, we used the shorter term rPFS data from the 2019 publication [6], consistent with our PFS definition.

# S3 Text. Modelling Survival and Progression of metastatic disease.

*Meta-analysis and extrapolation of the ADT survival curves*

We fitted a Cox proportional hazards (PH) model with a fixed treatment effect incorporating the re-created individual patient data (IPD) across all treatment strategies. For the ADT strategy only, we predicted pooled Kaplan Meier (KM) survival estimates from the overall Cox model until the longest trial observation period available (9 years) and extrapolated up to 15 years with the best-fitting parametric distribution for the pooled ADT data thereafter. We hence constructed piecewise survival curves for OS and PFS. We tried the most common parametric distributions for the OS and PFS curves (gamma, generalized gamma, lognormal, loglogistic, Weibull, Gompertz, exponential). Survival curves with the best fit for both OS and PFS were identified, based on the Akaike and Bayesian information criterion (AIC, BIC) but also based on clinical plausibility of 30-year extrapolations in terms of OS and PFS outcomes after 9 years.

*Comparator survival curves*

For each included study, the PH assumption between the survival for the comparator and the intervention was verified both visually and based on the Schoenfeld test (S5 Fig).

In case the PH-assumption was not substantially violated, we obtained survival curves of the intervention strategies by applying hazard ratios (HRs) of the Cox model to the ADT baseline hazard.

We used different HRs for the base case and scenario analyses:

- Base case: HRs from our meta-analysis of re-created IPD (excluding the three studies LATITUDE, ENZAMET, and NCT02058706).

For the docetaxel and the abiraterone part of the STAMPEDE study, we assumed that patients were from the same study, although both study parts only partially overlapped in time and hence only partially included the same patients to receive ADT.

- Scenario analyses: HRs from a modified Cox-model model and from different network meta-analyses (NMA))

1. Addition of a shared frailty term (for trials) in the base case model for the determination of the HRs.
2. HRs from our NMA by Menges et al. [7] based on aggregated data from the literature and excluding the three studies (LATITUDE, ENZAMET, NCT02058706)
3. As 2) but including the three studies (LATITUDE, ENZAMET, NCT02058706)
4. HRs from the NMA by Wang et al. [8]

Estimated survival curves for all strategies were converted into transition probabilities as one minus the ratio of the survivor function at the end and the beginning of a model cycle.

# S4 Text. Treatment strategies.

We assumed an equal distribution among the three agonists Leuprorelin (11.25mg, subcutaneous (sc.) every 3 months), Goserelin (10.8 mg, sc. every 3 months) and Triptorelin (11.25 mg, intramuscular injection every 3 months). The model also integrated additional administration of the oral anti-androgen bicalutamide (50 mg/day for 30 days) for all three agonists during the first month of treatment.

For osteoporosis prevention during ADT monotherapy and ADT combination strategies, we assumed oral calcium and vitamin D supplements would be given to all patients (e.g. Calcimagon® D3 Forte, 1 tablet per day unlimited).

The model also integrated one sc. injection every 6 months of denusumab (60 mg/ml) for a quarter of the patients in the ADT and ADT + docetaxel strategies, and for half of patients in the ADT + androgen receptor axis-targeted therapies (ARATs), based on medical expert opinion.

For the prevention of neutropenia due to docetaxel treatment, we assumed 6 mg Pegfilgrastim sc. injection 24 hours after each chemotherapy. We assumed administration to all patients on docetaxel. For these patients, we also included antiemetic treatment with both 16 mg dexamethasone and 8 mg ondansetron, once in each chemotherapy cycle.

# S5 Text. Adverse effects.

For the health economic model, we selected adverse effects (AEs) of severity grades 3-5, which we considered important based on the opinions of involved Swiss medical experts and given their potential of leading to hospitalizations. The use of AE grades 3-5 (including AE-related deaths), instead of grades 3-4, was necessitated by the limited reporting in some trial publications and may have led to an overestimation of AE rates. However, fatal (grade 5) AEs were infrequent in the trials, so that we do not expect them to have strongly influenced the overall AE rates for the described outcomes. We only considered adverse AEs until disease progression, but not for treatment lines thereafter.

Monthly AE incidence rates for each adverse event type were derived from our meta-analysis of AEs [7] in the form of monthly incidence rates for patients receiving ADT alone and treatment-specific rate ratios for the other treatment strategies (S2 Table). Incidence rates per trial arm and per AE type were calculated as the number of persons with AE events for each treatment group divided by the person-time at risk (i.e., the total number of participants per treatment group multiplied by the median follow-up time). From these estimates, we then estimated incidence rates for the ADT group across all trials as well as incidence rate ratios for each of the combination treatments by calculating a weighted mean estimate.

The AE calculation was limited by the reporting in some of the relevant trials (i.e., for each individual AE type, trials typically reported the AE of highest severity if multiple AEs of the same type occurred in a patient). In consequence, the resulting estimates of monthly incidence rates may be false low.

Again, the LATITUDE, ENZAMET, and NCT02058706 trials were excluded, while the CHAARTED, GETUG, STAMPEDE, ARCHES, and TITAN trials were included (depending on the coverage of the specific AE type in the publications).

# S6 Text. Further line treatments.

Further lines of treatment and their utilization were based on published literature, but also specified through consultation with two medical experts.

After ADT treatment for mHSPC patients, we assumed castration-resistant prostate cancer (CRPC) 1L monotherapy treatment with either abiraterone or enzalutamide (50% each), followed by CRPC 2L docetaxel (S3 Table). After ADT+docetaxel, we also modelled CRPC 1L abiraterone or enzalutamide monotherapy, but followed by CPRC 2L cabazitaxel treatment. After ADT+ARATs (abiraterone, enzalutamide, or apalutamide), our model assumed chemotherapy with docetaxel as CRPC 1L and with cabazitaxel as CRPC 2L. This was reported as advantageous in comparison to a further but different hormonal treatment (demonstrated by de Wit et al. 2019) [9]. Patients were assumed to receive cabazitaxel 20 mg/m2 intravenous (i.v.) every 3 weeks plus prednisone 10 mg daily. Radium-223 as a further-line treatment for a possibly small percentage of patients with bone metastasis was not included [9]. Cabazitaxel treatment was assumed until the reported median PFS time, which we defined in a simplified manner as the end of treatment time in our model. Furthermore, antiemetic treatments with 16 mg dexamethasone and 8 mg ondansetron once in each chemotherapy cycle were included as was pegfilgrastim for neutropenia prophylaxis under CRPC 2L cabazitaxel (the latter however only for half of the patients due to the cabazitaxel dose of 20 mg/m^2^).

With regard to the number of patients receiving CRPC 1L and 2L treatment, we modelled 90% of progressed patients to receive further line CRPC 1L treatment (feedback from two Swiss medical experts, international literature like e.g. Clarke et al. [1]) independent of their 1L strategy. For example in the docetaxel part of the STAMPEDE trial [1] 90% of progressed patients received CRPC 1L treatment after ADT, and 85% after ADT+docetaxel. We integrated CRPC 2L treatment for 32% of all patients (medical expert feedback, Akaza 2018 [10] 288/903=32%). CRPC 3L was not incorporated in the model due to the low number of published patients (9%) with such a treatment reported by the same authors [10].

We summarized distributional assumptions about post progression treatments in S4 Table. With regard to the durations of CRPC 1L and CRPC 2L treatments, we used their median PFS times as outlined in S5 Table.

All derived inputs for further line treatments were then entered into the TreeAge model which used tunnel states to remember for all sub-fractions of the modelled cohorts when they have entered a given health state. On this basis, treatment costs and utilities can be modelled dependent on the time already spent in the respective health state.

# S7 Text. End of life hospitalization costs.

For terminal / end of life (EoL) hospitalization costs, we applied a 2018 estimate of the Cantonal Hospital of Lucerne of CHF 22,816 [11], which we considered representative of the costs in Switzerland. Since the Swiss consumer price indices for the end of 2018 and 2021 were similar (101.5 and 102.4) [12], we only multiplied this rough cost estimate by the estimated percentage of EoL prostate cancer patients in Switzerland who are hospitalized (65%), without further adjustment for inflation. One-off EoL costs of CHF 22,816*0.65 = CHF 14,830 resulted per patient, corresponding to EUR 9,180, considering purchasing power parities of 0.619 [13].

# S8 Text. Uncertainty analyses.

In the deterministic sensitivity analyses, available 95% confidence intervals (CIs) were used as the maximum and minimum boundaries, or the 2.5% and 97.5% percentiles of assigned distributions (next paragraph). If both could not be determined, we varied the base case parameter value by ±30%. We presented the results in Tornado diagrams.

For the probabilistic sensitivity analyses (PSA), we assigned gamma distributions to unit cost parameters (to prevent values less than zero from being drawn), and beta distributions to utilities and probabilities. The beta distribution restricts draws to the 0-1 space. Distribution parameter estimated to represent the OS and PFS curves were assigned normal distributions, and HRs were assigned log-normal distributions. Where standard error estimates or 95% CIs were not available, we assumed standard errors to be 20% of the base case parameter values for costs and probabilities, and 10% for utilities. For AE disutilities, we assigned normal distributions with mean 1 and standard deviation 0.153, resulting in variation of the fix parameter by ±30%. We performed 10,000 simulation runs and showed the 10,000 simulated incremental cost-effectiveness ratios (ICERs) results in a cost-effectiveness plane. Strategies’ probabilities of being cost-effective at varying willingness-to-pay (WTP) thresholds were additionally illustrated in cost-effectiveness acceptability curves (CEACs).

AE costs were not varied individually per AE type in deterministic sensitivity analysis, but by treatment strategy. Also, the OS and PFS KM estimates of the ADT strategy could not be varied directly. The impact of their variation was examined by using the lower and upper 95% CIs of the KM estimates in two scenario analyses. All scenario analyses are outlined in S11 Table.

# S9 Text. Survival analysis results.

Fig. 1 and Supplementary Fig. A2 show the digitized OS and PFS KM curves under ADT treatment from all matching RCTs. KM OS curves from ENZAMET and LATITUDE were also presented for completeness and comparison purposes only, but were not included into the meta-analyses. In addition, we displayed the OS curve for ADT which was used in the cost-effectiveness analysis of Woods at al. [14], comparing ADT+docetaxel with ADT monotherapy. The re-created KM OS curves show that high-risk patients from LATITUDE seem to obtain lower OS values as our overall study population. In contrast, the survival of the high percentage of low-risk patients in the subgroup of patients without addition of docetaxel in ENZAMET seems to be higher. The OS and PFS graphs also demonstrate the immature data of the ARCHES trial, resulting in a sharp drop in the KM PFS curve after 2 years of follow-up (S2 Fig).

Supplementary Fig. A3 shows the pooled and extrapolated ADT OS and PFS curves (from the Cox model and a gamma or a loglogistic extrapolation after 9 years). A loglogistic parameterization led to the lowest AIC and BIC during the longest observation period available, but estimated OS values were well above zero after 20 years which we did not consider as realistic. We hence selected a gamma distribution as the steepest descending curve after 9 years for ADT, and investigated a loglogistic extrapolation in a scenario analysis. Finally, Fig. 2 and S4 Fig show the estimated OS and PFS curves for ADT and all intervention strategies.

Based on visual inspection of the re-created KM curves and the Schoenfeld plots, we considered the PH assumption to not be violated for OS and not substantially violated for PFS (Schoenfeld plots and p-values for PFS are shown in S5 Fig). Thus, we considered the chosen approach of combining ADT survival curves with relative treatment effect estimates for OS and PFS to be appropriate.

The complete list of HR results from our meta-analysis of individually re-created OS and PFS data together with the HRs from the scenario analyses (e.g. different network meta-analyses) are presented in S13 Table.

# S1 Table. Studies considered for estimation of survival curves.

| **Trials for OS Endpoint** | **Trials for PFS Endpoint** | **PFS Endpoint** | **Inclusion** |
| --- | --- | --- | --- |
| **ADT+docetaxel vs. ADT** |  | PFS | Yes |
| GETUG (Gravis et al. 2016) [15] | GETUG (Gravis et al., 2013) [16] | cPFS | Yes |
| CHAARTED (Kyriakopoulos et al., 2018)) [17] | CHAARTED (Sweeney et al., 2015) [18] | TT clinical progression | Yes |
| STAMPEDE (Clarke et al. 2019) [1] | STAMPEDE (Clarke et al. 2019) [1] | PFS | Yes |
| **ADT+abiraterone vs. ADT** |  |  |  |
| LATITUDE (Fizazi et al., 2019) [19], high-risk patients only available) | LATITUDE ((Fizazi et al., 2017) [20], only for high-risk subgroup available) | PFS | No |
| STAMPEDE (James et al., 2017) [21] results for M1 subgroup | STAMPEDE (James et al., 2017) [21] results for M1 subgroup | FFS | Yes for OS, but not for PFS |
| **ADT+enzalutamide vs. ADT** |  |  |  |
| ENZAMET ((Davis et al., 2019) [22], subgroup results of patients without docetaxel) | ENZAMET ((Davis et al., 2019)[22], subgroup results of patients without Doc) | cPFS | No |
| ARCHES (Armstrong et al., 2019) [23] | ARCHES (Armstrong et al., 2019)[23] | rPFS | Yes |
| NCT02058706 (Vaishampayan et al., 2021) [4] | NCT02058706 (Vaishampayan et al., 2021) [4] | bPFS | No |
| **ADT+apalutamide vs. ADT** |  |  |  |
| TITAN (Chi et al., 2021) [24] | TITAN (Chi et al., 2019) [25] | rPFS | Yes |

ADT, androgen-deprivation therapy; PFS, progression-free survival; bPFS, biological progression-free survival; cPFS, clinical progression-free survival; rPFS, radiographic progression-free survival; OS, overall survival.

# S2 Table. Adverse effect monthly rates for ADT and monthly incidence rate ratios for combination therapies.

| **Adverse effect** | **ADT AE rate** | **ADT+Doc vs ADT IRR** | **ADT+Abi vs ADT IRR** | **ADT+Apa vs ADT IRR** | **ADT+Enza vs ADT IRR** |
| --- | --- | --- | --- | --- | --- |
| Febrile neutropenia | 0.00012702 | 13.5259 | 2.0253 | 1 | 1 |
| Cardiac failure | 0.00008297 | 1.0051 | 1.0127 | 1 | 1.2042 |
| Cardiac arrhythmia | 0.00025868 | 1.0000 | 7.0886 | 1 | 0.4460 |
| Ischemic heart disease | 0.00043501 | 1.0207 | 1.1252 | 4.2240 | 0.5017 |
| Cerebrovascular disease | 0.00010681 | 1.0000 | 2.0253 | 5.5315 | 0.5017 |
| Fracture | 0.00013606 | 1.0000 | 1.0127 | 5.2801 | 1.0035 |

Abi, abiraterone; ADT, androgen deprivation therapy; AE, adverse effect; Apa, apalutamide; Doc, docetaxel; Enza, enzalutamide; IRR, incidence rate ratio; vs, versus.

# S3 Table. Post-progression treatments.

| **mHSPC treatment** | **CRPC 1L treatment^a^** | **CRPC 2L treatment^a^** |
| --- | --- | --- |
| ADT | 50% Abiraterone | Docetaxel |
|  | 50% Enzalutamide |  |
| ADT + Docetaxel | 50% Abiraterone | Cabazitaxel |
|  | 50% Enzalutamide |  |
| ADT + Abiraterone | Docetaxel | Cabazitaxel |
| ADT + Enzalutamide | Docetaxel | Cabazitaxel |
| ADT + Apalutamide | Docetaxel | Cabazitaxel |

1L, first-line; 2L, second-line.

^a^ Late-stage palliative care is also possible for some patients instead of further treatment lines. Percentage are described in detail in S4 Table.

# S4 Table. Proportion of patients with CRPC 1L, CRPC 2L, and late-stage palliative care treatment.

| **Further line treatments** | **Timepoint** | | |
| --- | --- | --- | --- |
|  | **Start CRPC 1L treatment** | **Start CRPC 2L treatment** | **End CRPC 2L treatment** |
| CRPC 1L and CRPC 2L | 90% CRPC 1L [0.34, 1]^a^ | 32% CRPC 2L [0.2, 0.45]^a^ | 32% new late-stage palliative |
| CRPC 1L, no CRPC 2L |  | 58% new late-stage palliative | Difference to 100% continues late-stage palliative |
| no CRPC 1L, no CRPC 2L | Difference to 100% is new late-stage palliative | Difference to 100% continues late-stage palliative |  |

CRPC, castration-resistant prostate cancer; PSA, probabilistic senstitivity analysis; 1L, first-line; 2L, second-line

^a^ In the PSA, the parameter was varied with a beta distribution (mean, mean*0.2). In deterministic sensitivity analysis we used the 2.5% and 97.5% percentiles of the beta distribution.

# S5 Table. CRPC first-line and CRPC second-line treatment durations.

| **Drug** | **Dosages** | **Median treatment duration^1^** | **PFS of further line (in months)^1^** | **Source** |
| --- | --- | --- | --- | --- |
| Abiraterone + prednisone 5mg | CRPC dosing: Abiraterone 1,000 mg + Prednisone 2x5 mg daily p.o. | = PFS duration of 5.5 | 5.5  (median radiologic PFS 5.6 months in publication) | HTA Fizazi et al. 2012 [26] |
| Cabazitaxel + prednisone 10mg | 20 mg/m^2^ as IV infusion every 21 days during median treatment duration | 22 weeks (5.2 months) | 8 | de Wit et al. 2019 [9] |
| Docetaxel + prednisone | 75 mg/m^2^ every 3 weeks as under 1^st^ line treatment but restricted to median treatment duration | 9.5 cycles of 3 weeks (6.6 months) | No PFD data was available in Collins et al. [27]. We hence assumed the same PFS as for Cabazitaxel: 8 months | Collins et al. HTA 2007 [27] |
| Enzalutamide | 160 mg once daily | = PFS duration of 5.5 | 5.5  (5.4 months radiographic PFS in publication) | NICE HTA [28] |

CRPC, castration-resistant prostate cancer; HTA, health technology assessment; mg, milligram; PFS, progression-free survival; p.o., orally; PSA, probabilistic sensitivity analysis; SA, sensitivity analysis; sd, standard deviation.

^1^ Values for PSA varied with a gamma distribution (mean, sd=mean*0.2). In deterministic SA, we used the 2.5% and 97.5% percentile of the gamma distribution.

Footnote: 1 Month = 30.44 days = 4.35 weeks, 1 year = 52.2 weeks.

# S6 Table. Routine care and monitoring resource use.

| **Treatment strategies** | **Routine care and monitoring resource use** | |
| --- | --- | --- |
|  | **Description Part 1** | **Part 2** |
| **ADT monotherapy** | Oncologist visit plus a set of laboratory tests every 3 months |  |
| **ADT + docetaxel** | 5 oncologist visits for 6 cycles + docetaxel i.v. administration + laboratory tests every 3 weeks for 6 cycles. One physician visit assumed together with ADT visit.  Pegfilgrastim (Neulasta®) administration every 3 weeks for 6 cycles. | ADT resource use needs to be added on top |
| **ADT + abiraterone** | Oncologist visit plus a set of laboratory tests every 2 weeks for 3 months. Afterwards every month. We assumed the 3-monthly physician visits always together with the ADT visit. |  |
| **ADT + enzalutamide, ADT + apalutamide** | Oncologist visit every 2 weeks for the first 3 months, and every month afterwards. Laboratory tests every month. 3-monthly visit and laboratory tests assumed together with ADT. |  |

ADT, androgen deprivation therapy; i.v., intravenous.

# S7 Table. Imaging resource use.

| **Imaging resource / procedure** | **Treatment strategy** | Frequencies | | | | | |
| --- | --- | --- | --- | --- | --- | --- | --- |
|  |  | Start | During 4-6 months | During all following 6 months | At progression / Start CRPC 1L (for 90% of patients) | During CRPC 1L (once during 4-6 months) | CRPC 1L-Progression (for 32% of the patients, once during first CRPC 2L cycle) |
| CT | ADT, ADT + docetaxel | 1 | 1 | 0 | 1 | 1 | 1 |
|  | ADT + ARAT | 1 | 1 | 1 | 1 | 1 | 1 |
| Scintigraphy | All strategies | 1 | 1 | 0 | 1 | 1 | 1 |
| Osteo- densitometry | All strategies | 1 | 0 | 0 | 0 | 0 | 0 |

ADT androgen deprivation therapy; ARAT androgen receptor axis-targeted therapy; CRPC castration-resistant prostate cancer; 1L first-line; 2L second-line.

# S8 Table. Drug and drug administration costs for mHSPC treatment.

|  | **Drug costs^1^** | | **Administration and laboratory costs^2^** | | | |  |
| --- | --- | --- | --- | --- | --- | --- | --- |
| **Treatments** | **Month specification** | **Monthly costs (in EUR)** | **Month** | **Monthly costs (in EUR)** | **Deterministic univariate sensitivity analysis** | **PSA distribution and SD variation** | **Pure monthly drug costs (EUR)** |
| ADT^3^ strategy | First month | 196 | Once every 3 months | 42 (125 every 3 months) | Variation of the fix parameter value (±30%) | Normal (mean=1, sd=0.153) |  |
|  | Following months | 102 |  |  |  |  |  |
| ADT^3^ + docetaxel^4^ strategy | First month | 1,972 | Months 1-4 | 392 |  |  | Docetaxel  574 |
|  | Months 2-4 | 1,878 |  |  |  |  |  |
|  | Months >=5 | 1,818 | Months >=5 | 0 |  |  |  |
| ADT^3^ + abiraterone^5^ strategy | First month | 911 | Months 1-3 | 238 |  |  | Generic abiraterone 704 |
|  | Following months | 817 | Months >=4 | 113 |  |  |  |
| ADT^3^ + enzalutamide^6^ strategy | First month | 2,508 | Months 1-3 | 203 |  |  | Enzalutamide  2,305 |
|  | Following months | 2,414 | Months >=4 | 137 |  |  |  |
| ADT^3^ + apalutamide^6^ strategy | First month | 2,538 | Months 1-3 | 203 |  |  | Apalutamide 2,334 |
|  | Following months | 2,443 | Months >=4 | 113 |  |  |  |
| Cabacitaxel^7^ | During median treatment duration | 3,404 |  | 375 |  |  | Cabazitaxel  2,928 |

ADT androgen deprivation therapy; EUR euros; LHRH luteinizing hormone-releasing hormone; PSA probabilistic sensitivity analysis; SD standard deviation.

^1^ costs are sourced from the Swiss specialty list [29] and include co-medication.

^2^ administration costs are based on TARMED [30] and the Swiss Analysis List [31].

^3^ incorporates bicalutamide for agonists and osteoporosis prevention.

^4^ incorporates antiemese, prednisone and neulasta.

^5^ incorporates prednisone and osteoporosis prevention.

^6^ incorporates osteoporosis prevention.

^7^ incorporates antiemese, prednisone and neulasta.

# S9 Table. Further mHSPC cost inputs.

| **Cost variables** | **Mean monthly costs (EUR)** | **Deterministic univariate sensitivity analysis** | | | **PSA distribution** | **Comment (DRG 2020 codes listed)** |
| --- | --- | --- | --- | --- | --- | --- |
|  |  | **Lower** | | **Upper** |  |  |
| **Adverse effect costs** |  |  | | |  |  |
| Febrile neutropenia | 3,555 | Combined AE pre-progression costs for each treatment arm were varied by ±30% | | | AE pre-progression costs overall for each treatment arm were varied with a Normal distribution (mean=1, sd=0.153) | Q60B, Q86B |
| Cardiac failure | 5,239 |  |  |  |  | F62C |
| Cardiac arrhythmia | 3,795 |  |  |  |  | F66B, F70B |
| Ischemic heart disease | 4,012 |  |  |  |  | F66B, F60B, F75D, F69B |
| Cerebrovascular disease | 6,276 |  |  |  |  | B70F, B70G, B69D |
| Fracture | 1,531 |  |  |  |  | I61B, I69C |
| **Imaging costs^1^** |  |  | | |  |  |
| Osteodensitometry | 41 | 26 | 58 | | Gamma (mean, sd= 0.2*mean) | TARMED 1.09 [30] |
| Computer tomography | 679 | 437 | 973 | |  | TAMED and material costs from Cantonal Hospital Sankt Gallen |
| Skeletal scintigraphy | 263 | 170 | 376 | |  | TARMED |
| **Late-stage palliative care costs^1^** |  |  | | |  |  |
| 8Gy Radio | 3,862 | 2,485 | 5,530 | | Gamma (mean, sd= 0.2*mean) | TARMED 1.09 (varied physician visits and palliative drug costs together in PSA) |
| Physician visit including laboratory tests | 125 | 76 | 175 | |  |  |
| Drugs | 70 | 43 | 98 | |  |  |
| **EoL** | 9,180 | 5,918 | 13,157 | |  | EOL estimate from the Cantonal Hospital of Lucerne [11,32] |

ADT, androgen deprivation therapy; AE, adverse effect; DRG, diagnosis related group; EoL, end-of life; EUR, euros; mHSPC, metastatic hormone-sensitive prostate cancer; PSA, probabilistic sensitivity analysis; SA, sensitivity analysis; SD, standard deviation.

^1^ Values for deterministic SA calculated as 2.5% and 97.5% percentiles of the PSA gamma distribution.

# S10 Table. Utility input parameters.

|  | **Mean** | **Adverse effect disutility duration (months)^2^** | **Deterministic univariate sensitivity analysis** | | **PSA distribution and SD variation** | **Source** |
| --- | --- | --- | --- | --- | --- | --- |
|  |  |  | **Lower** | **Upper** |  |  |
| **Progression-free health state** |  |  |  | |  |  |
| ADT | 0.83 | - | 0.6357^1^ | 0.9579^1^ | Beta (mean, sd=mean ±10%) | Sathianathen 2019 [33], Sung 2021 [34] |
| ADT + abiraterone | 0.865 | - | 0.6641 | 0.9822 |  | Sathianathen 2019 [33] based on LATITUDE [35], Sung 2021 [34] |
| ADT + apalutamide | 0.83 | - | 0.6372 | 0.9570 |  | Assumption (same as ADT alone), based on Chi 2019 [25] |
| ADT + enzalutamide | 0.83 | - | 0.6405 | 0.9579 |  | Assumption (same as ADT alone) |
| ADT + docetaxel (6 cycles) | 0.80 | - | 0.6217 | 0.9315 |  | Sathianathen 2019 [33] based on CHAARTED QoL results [36] |
| **Post-progression health state** |  |  |  | |  |  |
| Post-progression without terminal illness | 0.635 | - | 0.5060^1^ | 0.7520^1^ | Beta (mean ±10%) | Aguiar 2019 [37] (Post-progression hormone therapy utility=0.658, Post-progression docetaxel therapy utility=0.612) |
| Post-progression with terminal illness | 0.40 | - | 0.3225 | 0.4792 |  | After termination of 3L therapy, Heijnsdijk 2012 [38] |
| **Adverse effect utility decrements** |  |  |  | |  |  |
| Febrile neutropenia | -0.37 | 1 | Variation of the fix parameter value by ±30% | | Normal (Mean=1, sd=0.153). The combined AE disutilities including their length for each treatment arm were varied per treatment strategy | Sathianathen 2019 [16] |
| Cardiac failure | -0.14 | 12 |  |  |  | Utility for heart failure from Davies 2015 [21] |
| Cardiac arrythmia | -0.02 | 12 |  |  |  | Assumption (and based on Wehler [40] |
| Ischemic heart disease | -0.06 | 12 |  |  |  | Utility for myocardial infarction from Davies 2015 [39] |
| Cerebrovascular disease | -0.30 | 12 |  |  |  | Davies 2015 [39] |
| Fracture | -0.09 | 1 |  |  |  | Davies 2015 [39] (mean disutility of hip and arm fracture) |

ADT androgen deprivation therapy; AE adverse effect; mPC metastatic prostate cancer; PSA probabilistic sensitivity analysis; QoL quality of life; SD standard deviation; 3L third-line.

^1^ 2.5% and 97.5% percentiles of a beta distribution; similar for the utilities of the other treatments in this health state.

^2^ Durations of AE disutilities were not varied in sensitivity analyses.

# S11 Table. Scenario analyses.

| **No** | **Scenario** | **Description** |
| --- | --- | --- |
| 1 | HR NMA Menges et al. excluding ENZAMET/LATITUDE | HRs as outcomes of our NMA based on aggregated data from the literature and excluding LATITUDE and ENZAMET [7]. |
| 2 | HR NMA Menges et al. including ENZAMET/LATITUDE | HRs as outcomes of our NMA based on aggregated data from the literature and excluding LATITUDE and ENZAMET [7]. |
| 3 | HR NMA Wang et al. including ENZAMET/LATITUDE | HRs as outcomes of the NMA based on IPD (including LATITUDE and ENZAMET) [8]. |
| 4 | Loglogistic extrapolation of the survival curves | After the meta-analysis of the recreated IPD, extrapolation of the OS and PFS ADT curves with a loglogistic distribution instead of a gamma distribution (beyond the data cut-off for the trial with the longest observation period available (i.e., 9 years)). |
| 5 | Addition of shared frailty term in the model (for trials) for determination of HRs | Addition of a shared frailty term (for trials) next to a fixed treatment effect when determining the HRs of the intervention strategies. The random intercept allows death or PFS event under the reference strategy to vary across trials. |
| 6-7 | OS and PFS 95% CI under ADT treatment | Application of lower and upper 95% confidence intervals for the survival probabilities per cycle. |
| 8-9 | Discount rates | Discount rates for costs and QALYs: 0% and 5%. |
| 10-12 | Time horizon | Time horizon of 5, 10, and 15 years to explore the uncertainty of a large survival data extrapolation. |
| 13 | No continuous CT pre-progression for hormonal strategies | For ARAT-containing strategies during pre-progression, we assumed CT only at treatment start and 4-6 months afterwards; not every 6 months. Resource use during progression stayed the same. |
| 14 | No further line costs except EoL costs | We excluded further line drug acquisition, drug administration, imaging, and late-stage palliative radiotherapy costs except costs directly at progression. |
| 15 | EoL | Exclusion of EoL costs. |
| 16 | Originator abiraterone price | Monthly abiraterone generic drug costs (without co-medication costs) of EUR 704 instead of monthly drug costs with originator abiraterone of EUR 2,241 [29]. |
| 17 | Apalutamide monthly cost reductions of different percentages | Monthly apalutamide drug cost reduction by 67% (to EUR 770), 75% (to EUR 583), 80% (to EUR 467), and 90% (to EUR 233) instead of monthly originator costs of EUR 2,3334 (without co-medication costs). |
| 18 | Enzalutamide monthly cost reductions of different percentages | Monthly enzalutamide drug cost reduction by 67% (to EUR 761), 75% (to EUR 576), 80% (to EUR 461), and 90% (EUR 230) instead of monthly originator costs of EUR 2,305 (without co-medication costs). |

ADT, androgen deprivation therapy; ARAT, androgen receptor axis-targeted therapy; CI, confidence interval; CT, computed tomography; EoL, end of life; HR,  hazard ratio; IPD, individual patient data; NMA, network meta-analysis; OS, overall survival; PFS, progression-free survival; QALY, quality-adjusted life-year.

# S12 Table. Median survival results of recreated and pooled IPD.

| **Treatment strategies** | **Median^a^ (95% CI)**  **OS in months PFS in months** | |
| --- | --- | --- |
| ADT | 48.0 (45.0, 50.7) | 22.0 (20.1, 22.9) |
| ADT+docetaxel | 57.5 (53.8, 62.2) | 32.9 (29.3, 36.5) |
| ADT+abiraterone | 76.0 (65.2, 92.4) | - |
| ADT+apalutamide | 71.3 (62.2, 85.4) | 62.1 (4.6, 90.8) |
| ADT+enzalutamide | 76.9 (59.4, NA) | 69.2 (45.6, NA) |

ADT, androgen deprivation therapy; CI, confidence interval; IPD, individual patient data; NA, not applicable; OS, overall survival; PFS, progression-free survival.

^a^ Cox model included a fixed treatment effect.

Comment: Without data extrapolation beyond the clinical trial results.

# S13 Table. Hazard ratios of base case and scenario analyses.

| **OS** | **Doc (95% CI)** | **Abi (95% CI)** | **Apa (95% CI)** | **Enza (95% CI)** |
| --- | --- | --- | --- | --- |
| Recreated IPD excluding ENZAMET, LATITUDE, NCT02058706 (fixed treatment effect); base case | 0.81 (0.73, 0.90) | 0.60 (0.51, 0.72) | 0.64 (0.54,0.75) | 0.59 (0.43, 0.81) |
| Recreated IPD excluding ENZAMET, LATITUDE, NCT02058706 (fixed treatment effect and frailty term) | 0.81 (0.73, 0.90) | 0.60 (0.50, 0.71) | 0.65 (0.54, 0.77) | 0.61 (0.44, 0.84) |
| NMA of aggregated results from Menges et al. excluding ENZAMET, LATITUDE, NCT02058706)[7] | 0.77 (0.68, 0.88) | 0.65 (0.51, 0.85) | 0.65 (0.50, 0.83) | 0.81 (0.51, 1.29) |
| NMA of aggregated results from Menges et al. including all studies[7] | 0.77 (0.69, 0.85) | 0.66 (0.58, 0.74) | 0.65 (0.53, 0.79) | 0.59 (0.46, 0.77) |
| NMA of re-created IPD from Wang et al. including ENZAMET and LATITUDE[8] | 0.79 (0.71, 0.89) | 0.61 (0.54, 0.70) | 0.67 (0.51, 0.89) | 0.81 (0.53, 1.24) |
| **PFS** | **Doc (95% CI)** | **Abi (95% CI)** | **Apa (95% CI)** | **Enza (95% CI)** |
| Recreated IPD excluding ENZAMET and LATITUDE (fixed treatment effect); base case | 0.71 (0.64, 0.79) | Taken from the NMA since no matching PFS data was available when excluding LATITUDE | 0.49 (0.41, 0.58) | 0.46 (0.37, 0.57) |
| Recreated IPD excluding ENZAMET and LATITUDE (fixed treatment effect and frailty term) | 0.70 (0.63, 0.78) |  | 0.50 (0.40, 0.61) | 0.40 (0.31, 0.51) |
| NMA of aggregated results from Menges excluding ENZAMET and LATITUDE[7] | 0.67 (0.60, 0.74) | 0.45 (0.38, 0.54) | 0.48 (0.39, 0.60) | 0.39 (0.30, 0.50) |
| NMA of aggregated results from Menges et al. including all studies[7] | 0.67 (0.60, 0.74) | 0.46 (0.41, 0.52) | 0.48 (0.39, 0.60) | 0.36 (0.30, 0.44) |
| NMA of re-created IPD Wang et al. including ENZAMET and LATITUDE[8] | 0.67 (0.60, 0.74) | 0.51 (0.45, 0.58) | 0.48 (0.39, 0.60) | 0.39 (0.30, 0.50) |

Abi, abiraterone; Apa, apalutamide; CI, confidence interval; Doc, docetaxel; Enza, enzalutamide; IPD, individual patient data; NMA, network meta-analysis; OS, overall survival; PFS, progression-free survival.

# S14 Table. Base case lifetime cost results per cost component for each treatment strategy (discounted, in EUR).

| **Treatment costs** | **Drug acquisition & administration** (mean, % of total costs) | **Imaging** | **Adverse effects** | **Late-stage palliative care** | **Terminal care (EoL)** | **Total costs** |
| --- | --- | --- | --- | --- | --- | --- |
| ADT | 10,477 (33%) | 2,103 | 161 | 10,788 | 7,995 | 31,524 |
| ADT + docetaxel | 19 977 (51%) | 1,916 | 236 | 8,967 | 7,784 | 38,880 |
| ADT + abiraterone | 69,809 (75%) | 8,491 | 798 | 6,561 | 7,425 | 93,084 |
| ADT + apalutamide | 171,346 (88%) | 8,127 | 897 | 6.826 | 7,499 | 194,695 |
| ADT + enzalutamide | 177,496 (89%) | 8,473 | 190 | 6,949 | 7,411 | 200,519 |

ADT, androgen deprivation therapy; EoL, end-of-life; EUR, euros.

# S15 Table. Main scenario analysis results.

|  | **Description** | **ADT** | | **ADT + docetaxel** | | | **ADT + abiraterone** | | | **ADT + apalutamide** | | | **ADT + enzalutamide** | | |
| --- | --- | --- | --- | --- | --- | --- | --- | --- | --- | --- | --- | --- | --- | --- | --- |
| **No** |  | **Cost^2^** | **QALY^3^** | **Cost^2^** | **QALY^3^** | **ICER^1^** | **Cost^2^** | **QALY^3^** | **ICER^1^** | **Cost^2^** | **QALY^3^** | **ICER^1^** | **Cost^2^** | **QALY^3^** | **ICER^1^** |
|  | **Base case** | **31,524** | **3.25** | **38,880** | **3.97** | **10,205** | **93,084** | **5.33** | **39,814** | **194,695** | **4.90** | **D** | **200,519** | **5.15** | **D** |
| 1 | HR of NMA Menges et al. excluding the studies | 31,524 | 3.25 | 38,736 | 4.14 | 8,070 | 89,202 | 5.08 | 53,615 | 174,731 | 4.23 | **D** | 193,192 | 4.85 | **D** |
| 2 | HR of NMA Menges et al. including all studies | 31,524 | 3.25 | 38,981 | 4.16 | 8,203 | 89,322 | 5.08 | 54,345 | 193,554 | 4.86 | **D** | 211,689 | 5.35 | **456,383** |
| 3 | HR of NMA Wang et al. | 31,524 | 3.25 | 38,340 | 4.09 | 8,122 | 91,636 | 5.19 | 48,414 | 190,353 | 4.76 | **D** | 175,168 | 4.34 | **D** |
| 4 | Loglogistic extrapolation | 32,820 | 3.57 | 40,619 | 4.49 | 8,483 | 105,501 | 6.21 | 37,743 | 222,120 | 5.68 | **D** | 229,923 | 6.00 | **D** |
| 5 | Additional shared frailty model term for HR estimation | 31,524 | 3.25 | 38,566 | 3.98 | 9,612 | 98,657 | 5.37 | 39,740 | 192,671 | 4.85 | **D** | 203,778 | 5.16 | **D** |
| 6 | 95% LCLs for OS + PFS ADT curves | 31,128 | 3.06 | 38,542 | 3.73 | 10,966 | 88,737 | 5.03 | 38,811 | 183,901 | 4.62 | **D** | 189,418 | 4.85 | **D** |
| 7 | 95% UCLs for OS + PFS ADT curves | 31,960 | 3.47 | 39,236 | 4.25 | 9,434 | 98,036 | 5.68 | 41,036 | 207,061 | 5.22 | **D** | 213,161 | 5.48 | **D** |
| 8 | Discount rates 0% | 35,170 | 3.60 | 43,023 | 4.51 | 8,638 | 109,346 | 6.29 | 37,423 | 227,031 | 5.74 | **D** | 235,649 | 6.08 | **D** |
| 9 | Discount rates 5% | 29,560 | 3.06 | 36,708 | 3.68 | 11,395 | 84,867 | 4.85 | 41,288 | 178,202 | 4.47 | **D** | 182,771 | 4.68 | **D** |
| 10 | Time horizon 5 years | 22,888 | 2.37 | 28,978 | 2.62 | 24,453 | 54,603 | 3.05 | 59,442 | 116,762 | 2.88 | **D** | 116,810 | 2.94 | **D** |
| 11 | Time horizon 10 years | 28,953 | 3.04 | 35,435 | 3.56 | 12,476 | 77,830 | 4.44 | 48,226 | 165,381 | 4.14 | **D** | 167,551 | 4.28 | **D** |
| 12 | Time horizon 15 years | 30,969 | 3.21 | 37,944 | 3.86 | 10,675 | 87,683 | 5.02 | 43,055 | 184,787 | 4.65 | **D** | 188,778 | 4.84 | **D** |
| 13 | Less CT for hormonal strategies pre-progression | 31,524 | 3.25 | 38,880 | 3.97 | 10,205 | 86,368 | 5.33 | 34,881 | 188,372 | 4.90 | **D** | 193,842 | 5.15 | **D** |
| 14 | Exclusion of further line drug, admin, imaging, and late-stage palliative radiotherapy costs | 16,656 | 3.25 | 26,563 | 3.97 | 13,743 | 83,902 | 5.33 | 42,117 | 184,996 | 4.90 | **D** | 190,801 | 5.15 | **D** |
| 15 | Without EoL costs | 23,529 | 3.25 | 31,096 | 3.97 | 10,497 | 85,659 | 5.33 | 40,078 | 187,196 | 4.90 | **D** | 193,108 | 5.15 | **D** |
| 16 | Abiraterone originator price | 33,191 | 3.25 | 40,084 | 3.97 | 9,562 | 196,748 | 5.33 | **115,074** | 194,695 | 4.90 | **D** | 200,519 | 5.15 | **D** |

Admin administration; CT computed tomography; D dominated; EoL end of life; EUR euros; ICER incremental cost-effectiveness ratio; LCL lower confidence limit; QALY quality-adjusted life year; UCL upper confidence limit.

^1^ Comparison versus previous non-dominated strategies.

^2^ in EUR.

^3^ in costs per QALY gained.

# S16 Table.Scenario analysis results for apalutamide price reduction.

| **Scenario analysis** | **Result** | | | | |
| --- | --- | --- | --- | --- | --- |
| **Apalutamide 67% monthly price reduction to EUR 770** (without co-medication) | ADT+apalutamide absolutely dominated by ADT+abiraterone | | | | |
| **Apalutamide 75% monthly price reduction to EUR 583** (without co-medication) | ADT+apalutamide absolutely dominated by ADT+abiraterone | | | | |
| **Apalutamide 80% monthly price reduction to EUR 466.80** (without co-medication) | **Cost (EUR)** | **Incr Cost (EUR)** | **QALY** | **Incr QALY** | **ICER (EUR)** |
| ADT | 31,524 |  | 3.25 |  |  |
| ADT + docetaxel | 38,880 | 7,357 | 3.97 | 0.72 | 10,205 |
| ADT + apalutamide | 74,427 | 35,546 | 4.90 | 0.93 | 38,150 |
| ADT + abiraterone | 93,084 | 18,657 | 5.33 | 0.43 | 43,424 |
| **Apalutamide 90% monthly price reduction to EUR 233** (without co-medication) |  |  |  |  |  |
| ADT | 31,524 |  | 3.25 |  |  |
| ADT + docetaxel | 38,880 | 7,357 | 3.97 | 0.72 | 10,205 |
| ADT + apalutamide | 59,394 | 20,513 | 4.90 | 0.93 | 22,016 |
| ADT + abiraterone | 93,084 | 33,690 | 5.33 | 0.43 | 78,413 |

ADT, androgen deprivation therapy; EUR, Euro; ICER, Incremental cost-effectiveness ratio; Incr, Incremental; QALY, quality-adjusted life-year.

Comment: The table does not present results of dominated strategies

# S17 Table. Scenario analysis results for enzalutamide price reduction.

| **Scenario analysis** | **Result** | | | | |
| --- | --- | --- | --- | --- | --- |
| **Enzalutamide 67% monthly price reduction to EUR 761** (without co-medication) | ADT+enzalutamide absolutely dominated by ADT+abiraterone | | | | |
| **Enzalutamide 75% price reduction to EUR 576.18** (without co-medication) | **Cost (EUR)** | **Incr Cost (EUR)** | **QALY** | **Incr QALY** | **ICER (EUR)** |
| ADT | 29,635 |  | 3.25 |  |  |
| ADT + docetaxel | 37,516 | 7,881 | 3.97 | 0.72 | 10,933 |
| ADT + enzalutamide | 83,721 | 46,205 | 5.15 | 1.18 | 39,205 |
| ADT + abiraterone | 93,084 | 9,363 | 5.33 | 0.18 | 51,199 |
| **Enzalutamide 80% price reduction to EUR 461** (without co-medication) |  |  |  |  |  |
| ADT | 29,509 |  | 3.25 |  |  |
| ADT + docetaxel | 37,425 | 7,916 | 3.97 | 0.72 | 10,982 |
| ADT + enzalutamide | 75,934 | 38,509 | 5.15 | 1.18 | 32,675 |
| ADT + abiraterone | 93,084 | 17,149 | 5.33 | 0.18 | 93,779 |
| **Enzalutamide 90% price reduction to EUR 230** (without co-medication) |  |  |  |  |  |
| ADT | 29,257 |  | 3.25 |  |  |
| ADT + docetaxel | 37,243 | 7,986 | 3.97 | 0.72 | 11,079 |
| ADT + enzalutamide | 60,362 | 23,118 | 5.15 | 1.18 | 19,616 |
| ADT + abiraterone | 93,084 | 32,722 | 5.33 | 0.18 | 178,937 |

ADT, androgen deprivation therapy; EUR, Euro; ICER, Incremental cost-effectiveness ratio; Incr, Incremental; QALY, quality-adjusted life-year.

Comment: The table does not present results of dominated strategies


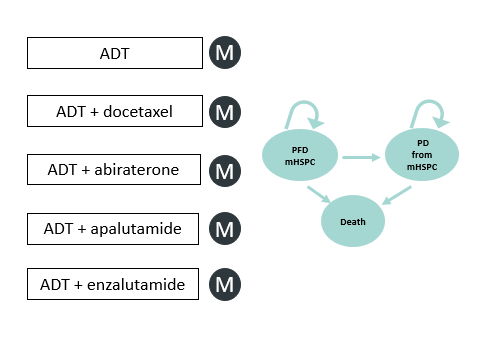


#

# S1 Fig. Markov model bubble diagram for the treatment of mHSPC.

ADT, androgen deprivation therapy; M, Markov model node; mHSPC, metastatic hormone-sensitive prostate cancer; PD, progressive disease; PFD, progression-free disease.


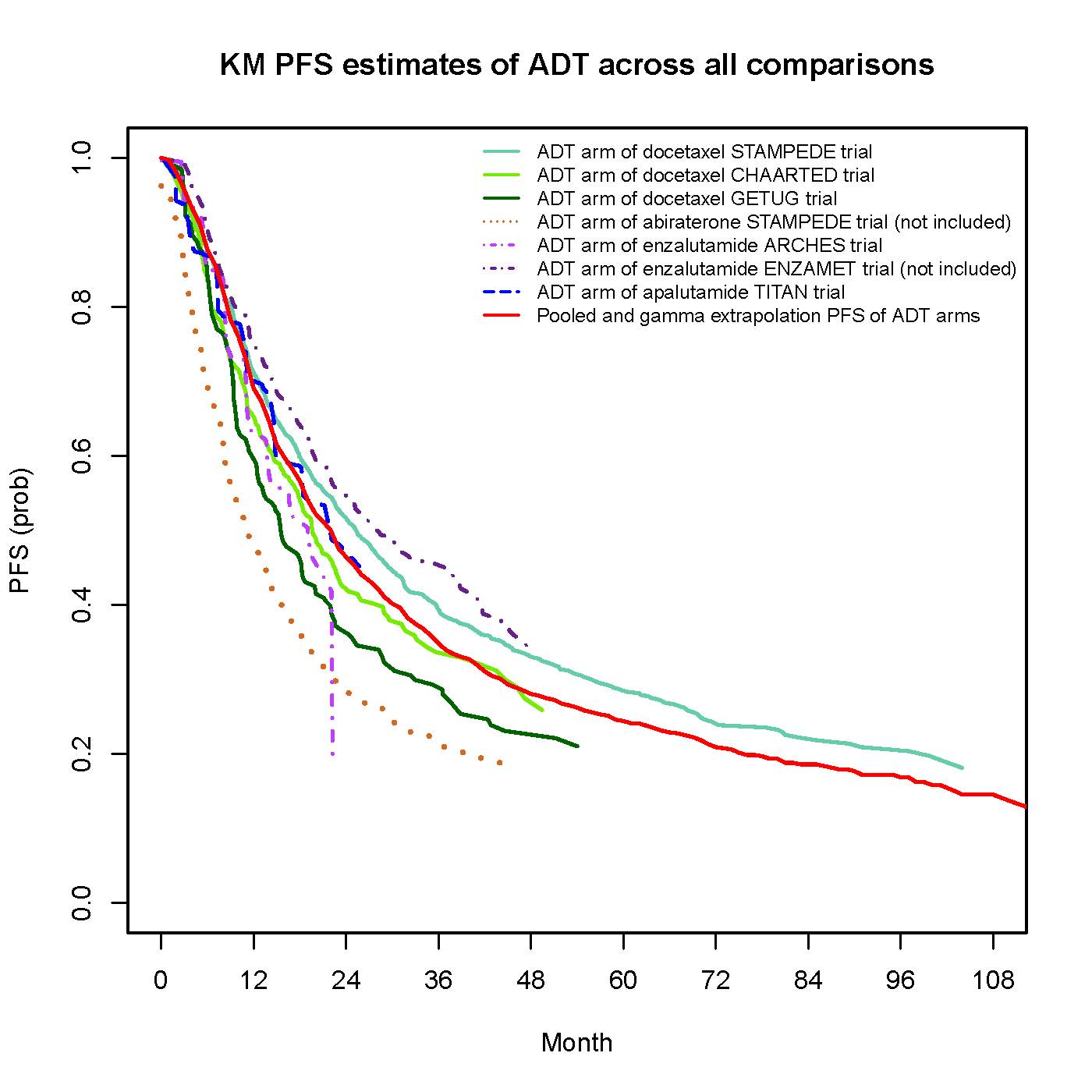


# S2 Fig. Pooled PFS curve for ADT and re-created Kaplan Meier PFS estimates for the ADT arms of each trial.

Abi, abiraterone; ADT, androgen deprivation therapy; Apa, apalutamide; Doc, docetaxel; Enza, enzalutamide; OS, overall survival.

Comment: ENZAMET and LATITUDE were not included in the meta-analysis.


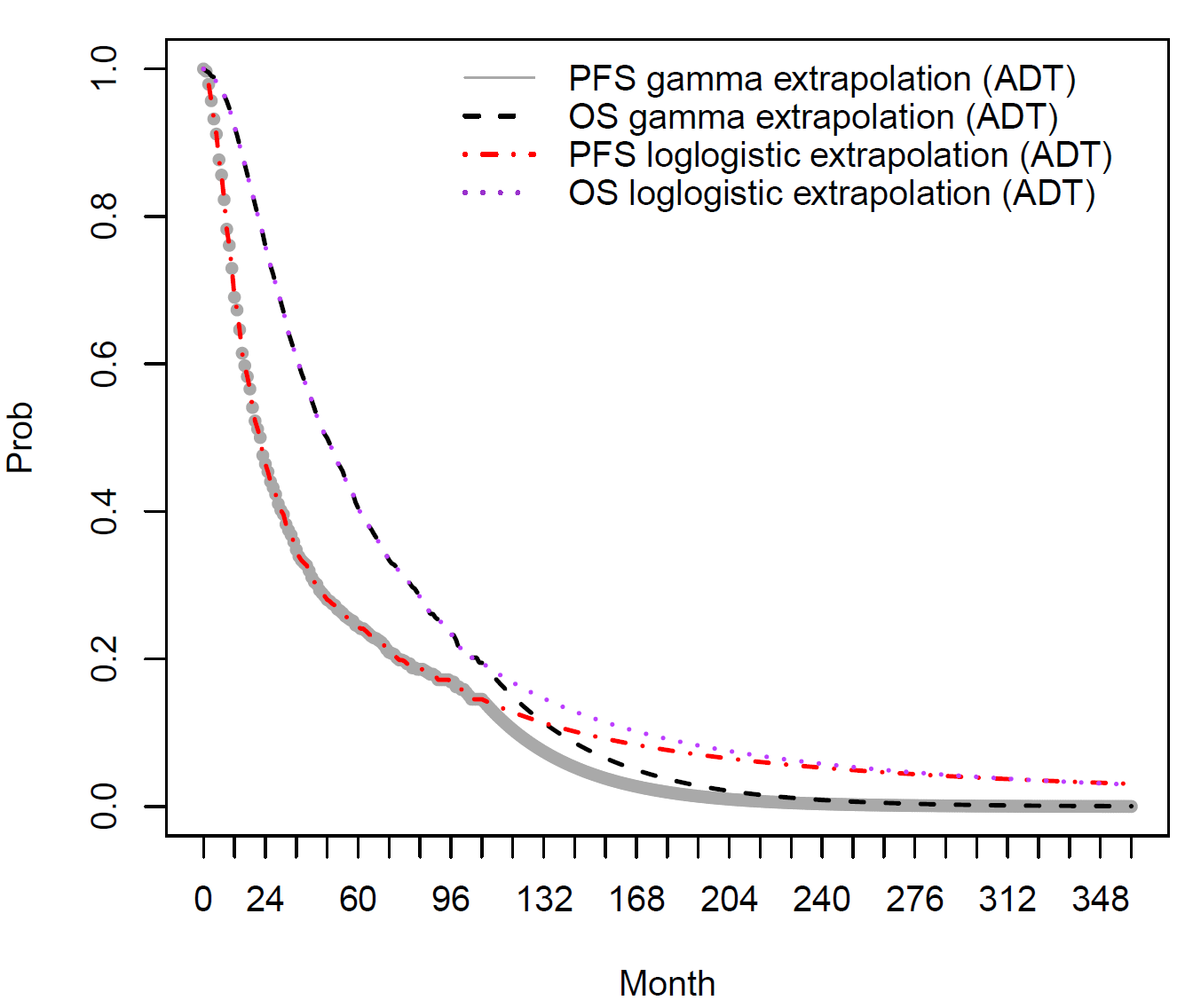


# S3 Fig. Pooled and extrapolated OS and PFS curves for ADT with re-created individual patient survival data.

ADT, androgen deprivation therapy; OS, overall survival; PFS, progression-free survival.


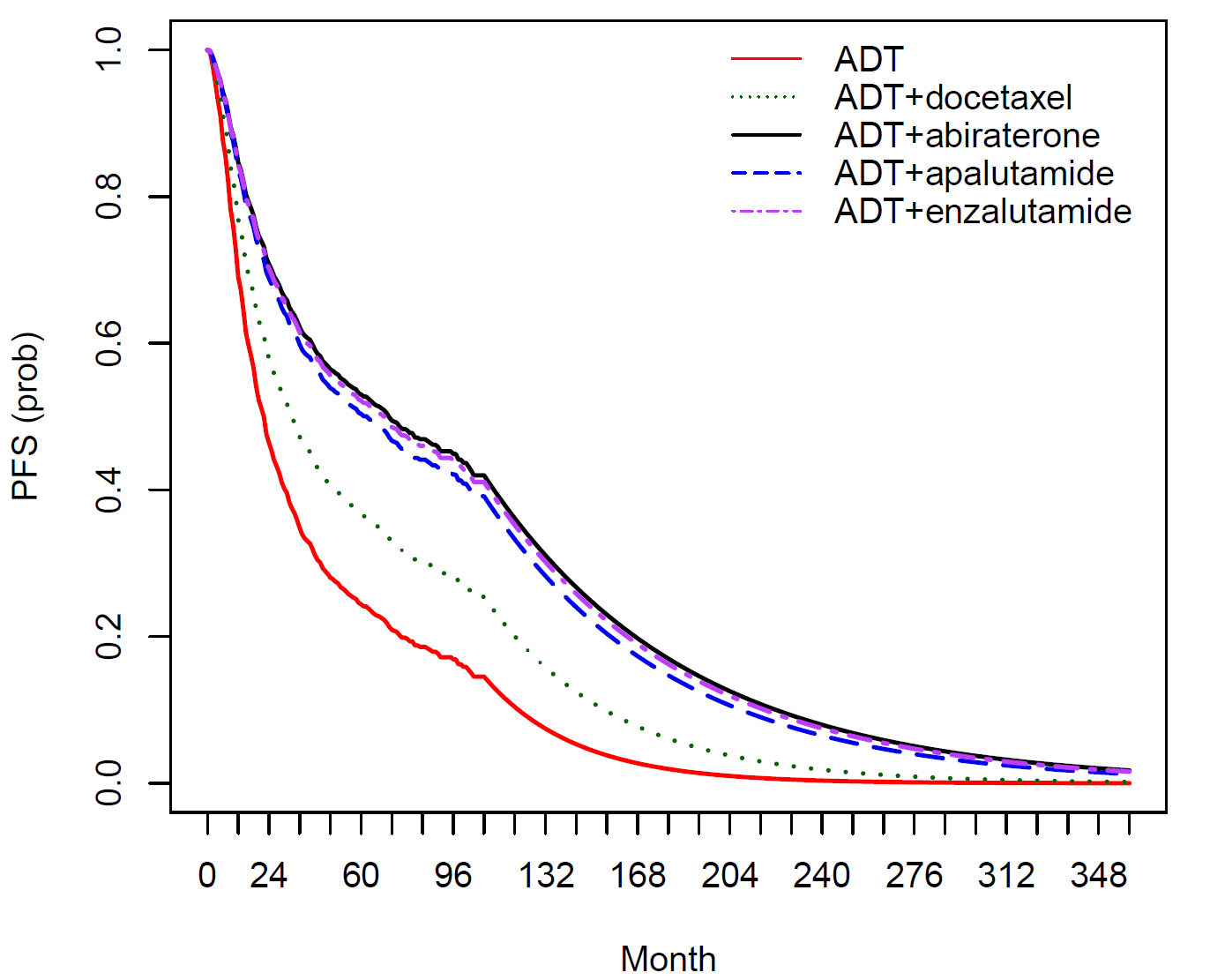


# S4 Fig. Estimated PFS curves for ADT and intervention strategies (gamma extrapolation).

ADT, androgen deprivation therapy; PFS, progression-free survival.

# S5 Fig. PFS PH assumption results (Schoenfeld test) and Kaplan-Meier curves from re-created individual patient data: (a) GETUG/Gravis 2013 (b) CHAARTED/Sweeney 2015 (c) STAMPEDE/Clarke 2019 (d) TITAN/Chi 2019 (e) ARCHES/Armstrong 2019.

**a** ADT+docetaxel versus ADT: PFS. GETUG/Gravis 2013 [41] (Schoenfeld test *p=0.0016*)


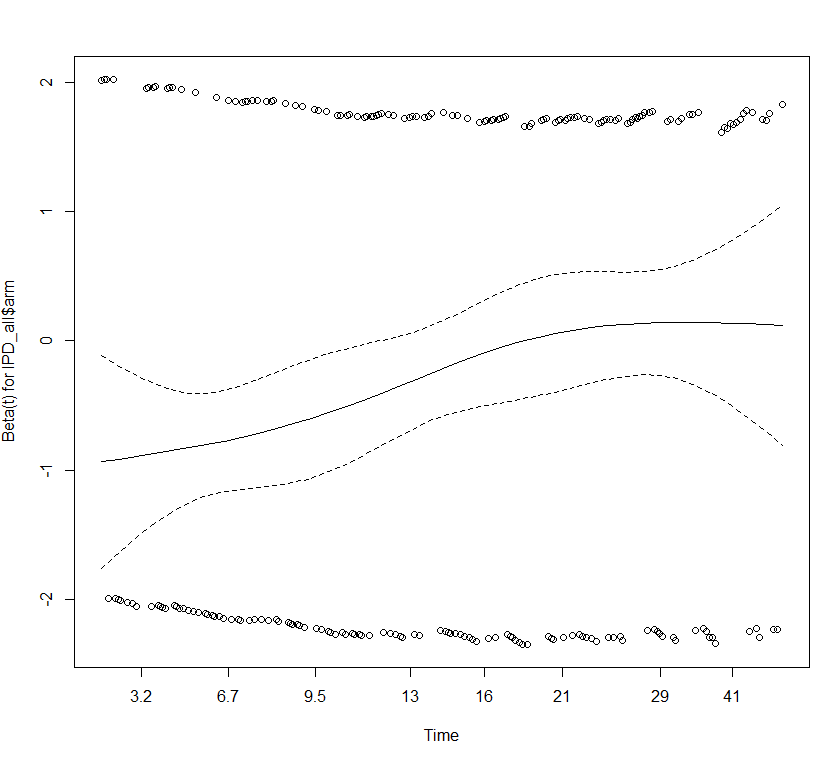


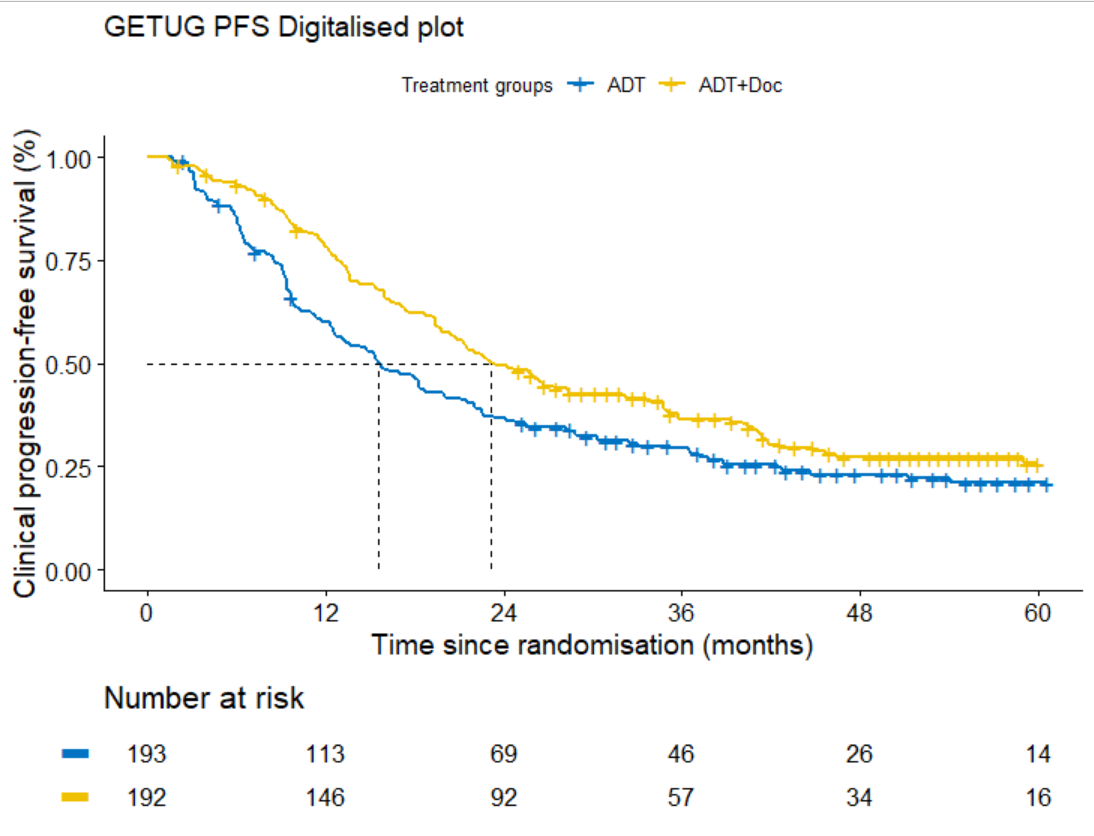


ADT, androgen deprivation therapy; Doc, docetaxel.

**b** ADT+docetaxel versus ADT: PFS. CHAARTED/Sweeney 2015 [18] (*p=0.0064*)


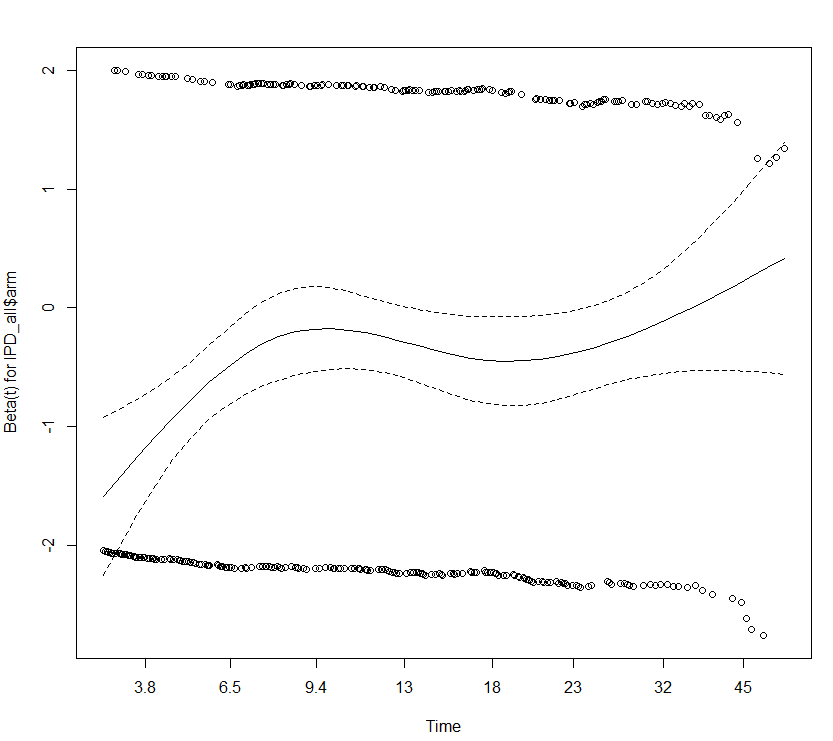


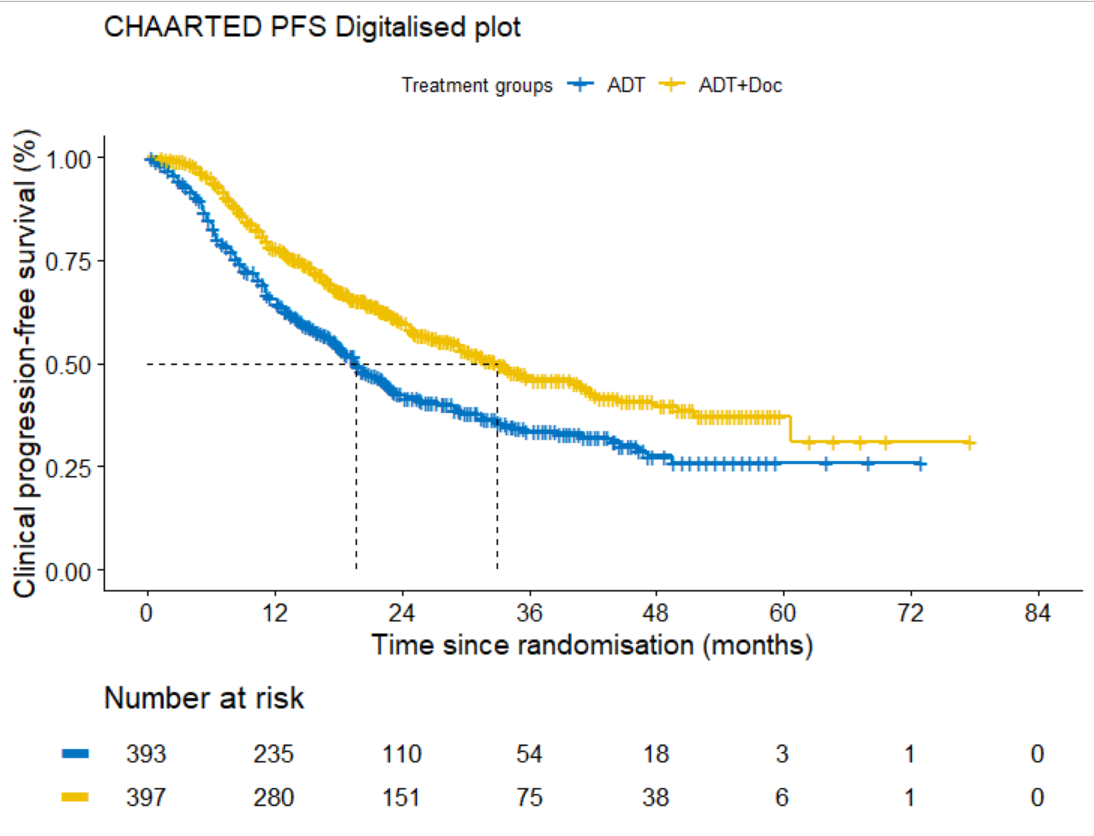


ADT, androgen deprivation therapy; Doc, docetaxel.

**c** ADT+docetaxel versus ADT: PFS. STAMPEDE/Clarke 2019 [1] (*p=0.0065*)


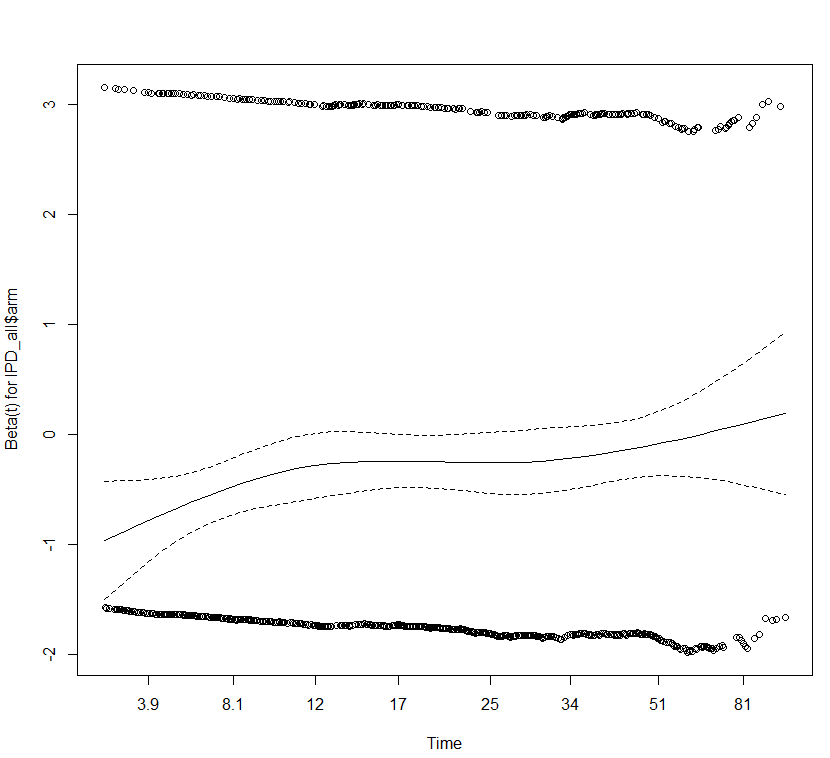


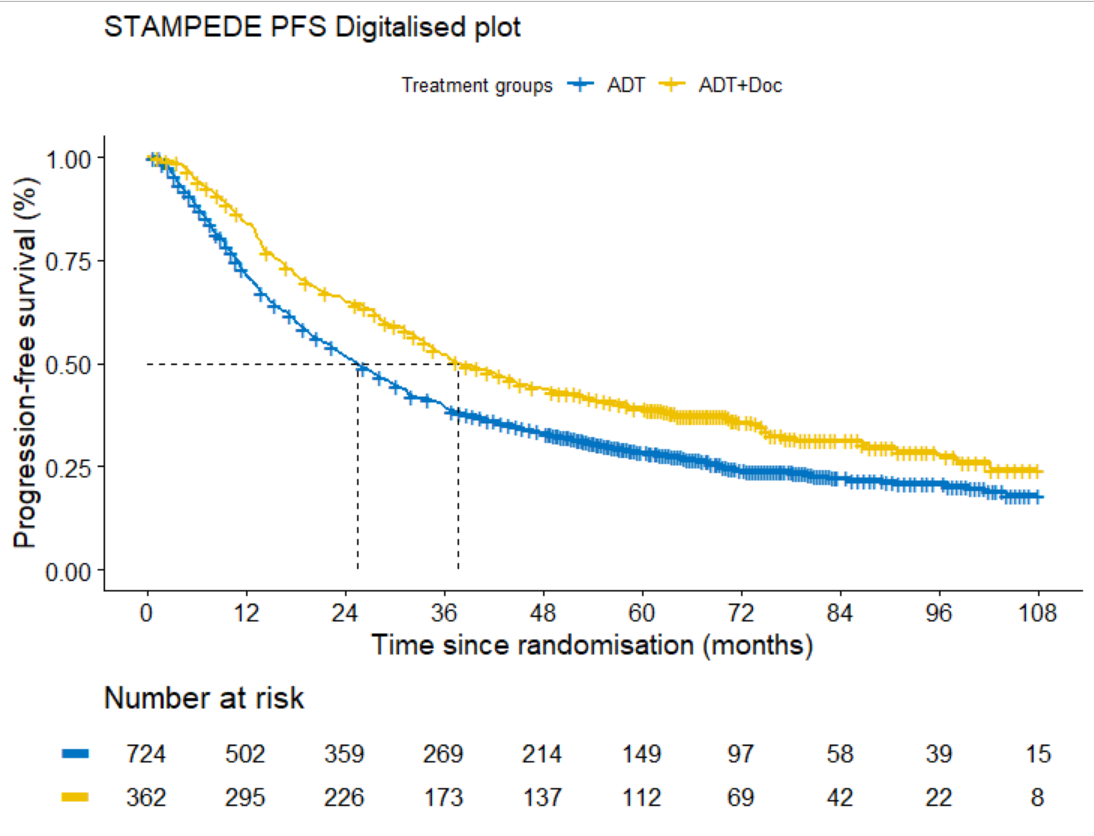


ADT, androgen deprivation therapy; Doc, docetaxel.

**d** ADT+apalutamide versus ADT: rPFS. TITAN/Chi 2019 [25] *(p=0.018)***
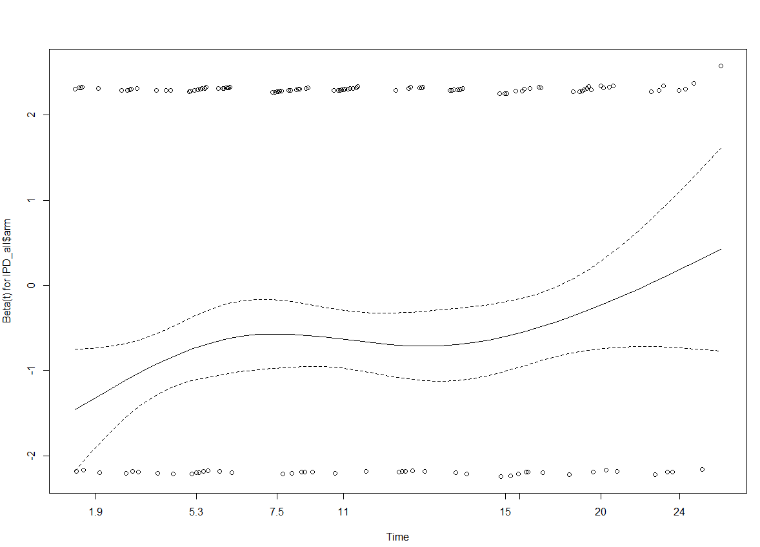
**


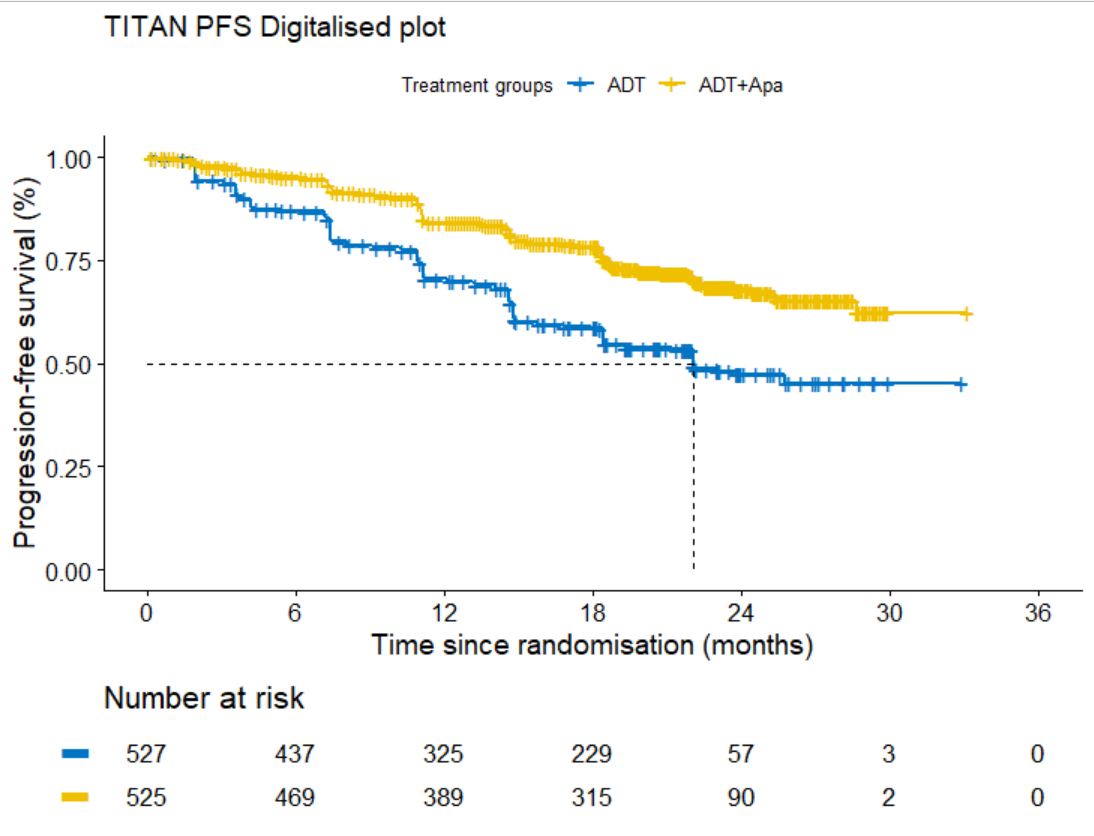


ADT, androgen deprivation therapy; Apa apalutamide.


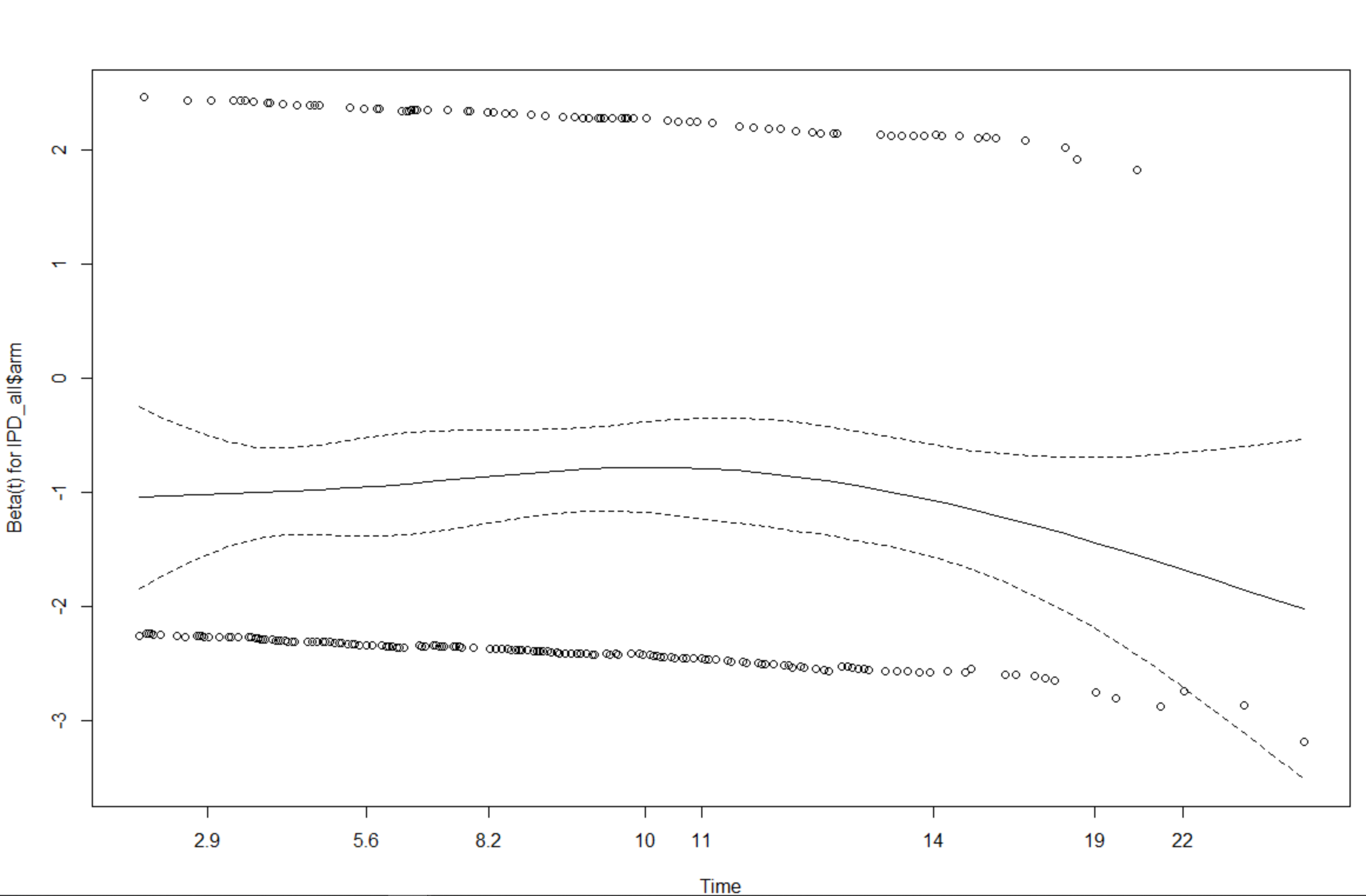
**e** ADT+enzalutamide versus ADT: rPFS. ARCHES/Armstrong 2019 [23] (*p=0.6053*)


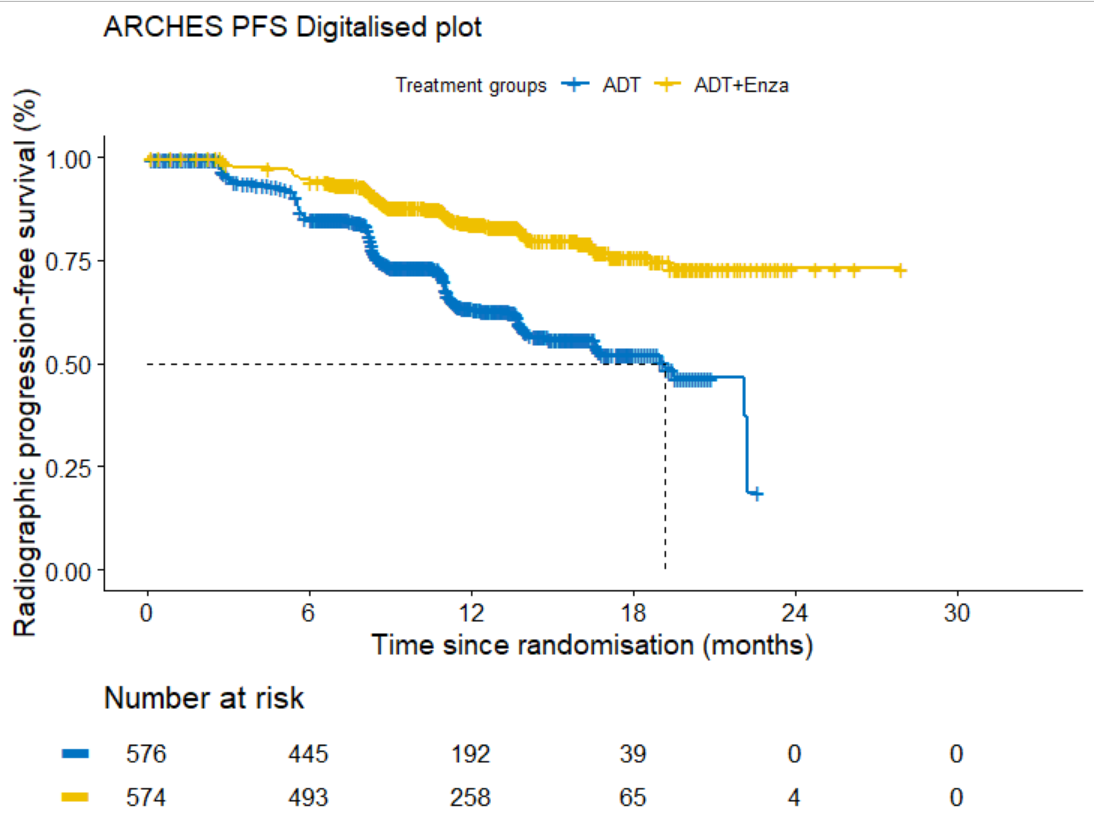


ADT, androgen deprivation therapy; Enza, enzalutamide.


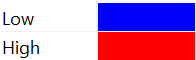

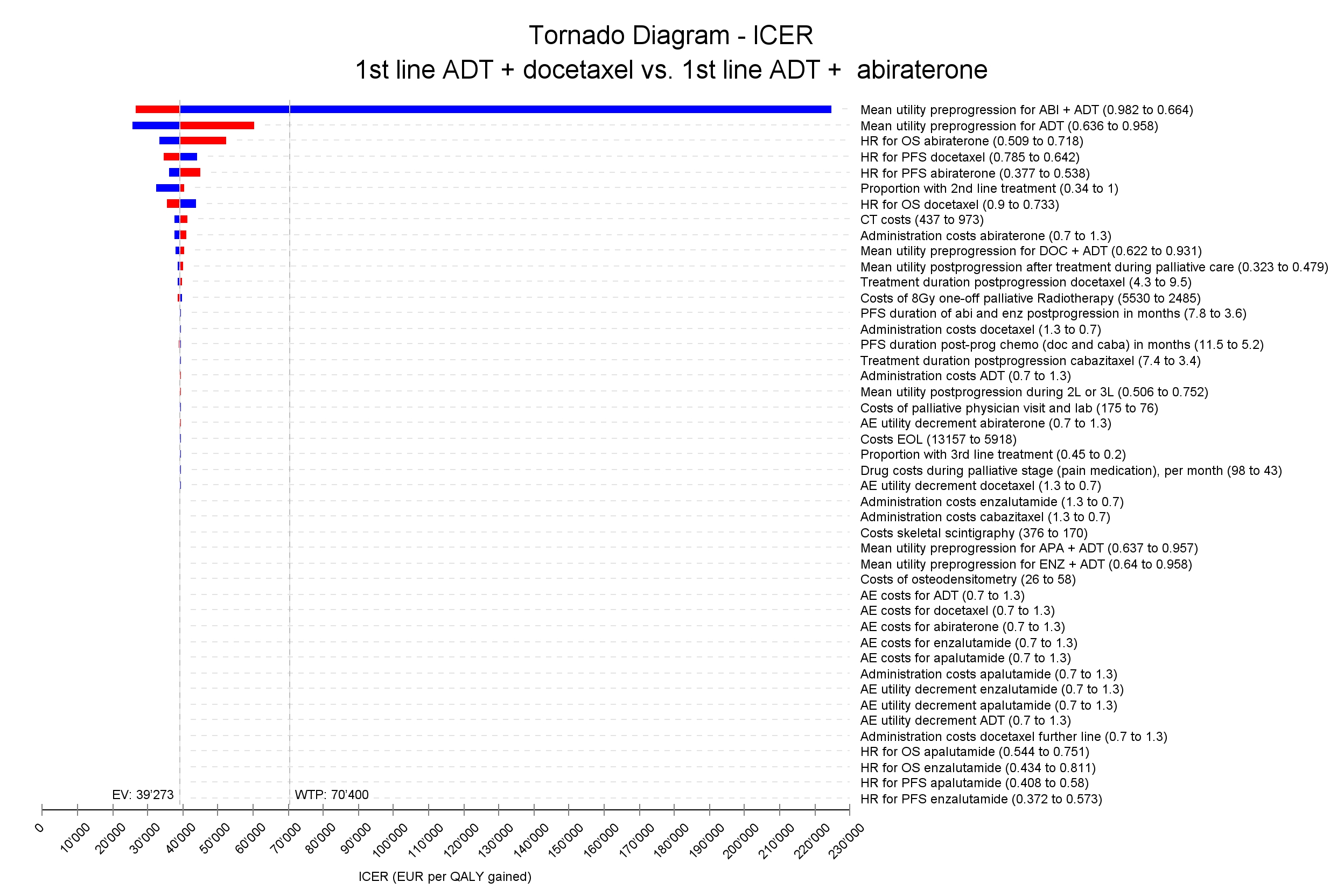


# S6 Fig. Tornado graph base case (ADT+docetaxel versus ADT+abiraterone).

ABI, abiraterone; ADT, androgen deprivation therapy; AE, adverse effect; APA, apalutamide; CRPC, castration resistant prostate cancer; CT, computed tomography; DOC, docetaxel; ENZ, enzalutamide; EOL,  end of life; EUR, euros; EV, expected value; HR, hazard ratio; ICER, incremental cost-effectiveness ratio; OS, overall survival; PFS, progression-free survival; WTP, willingness-to-pay.


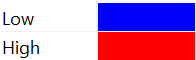


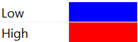

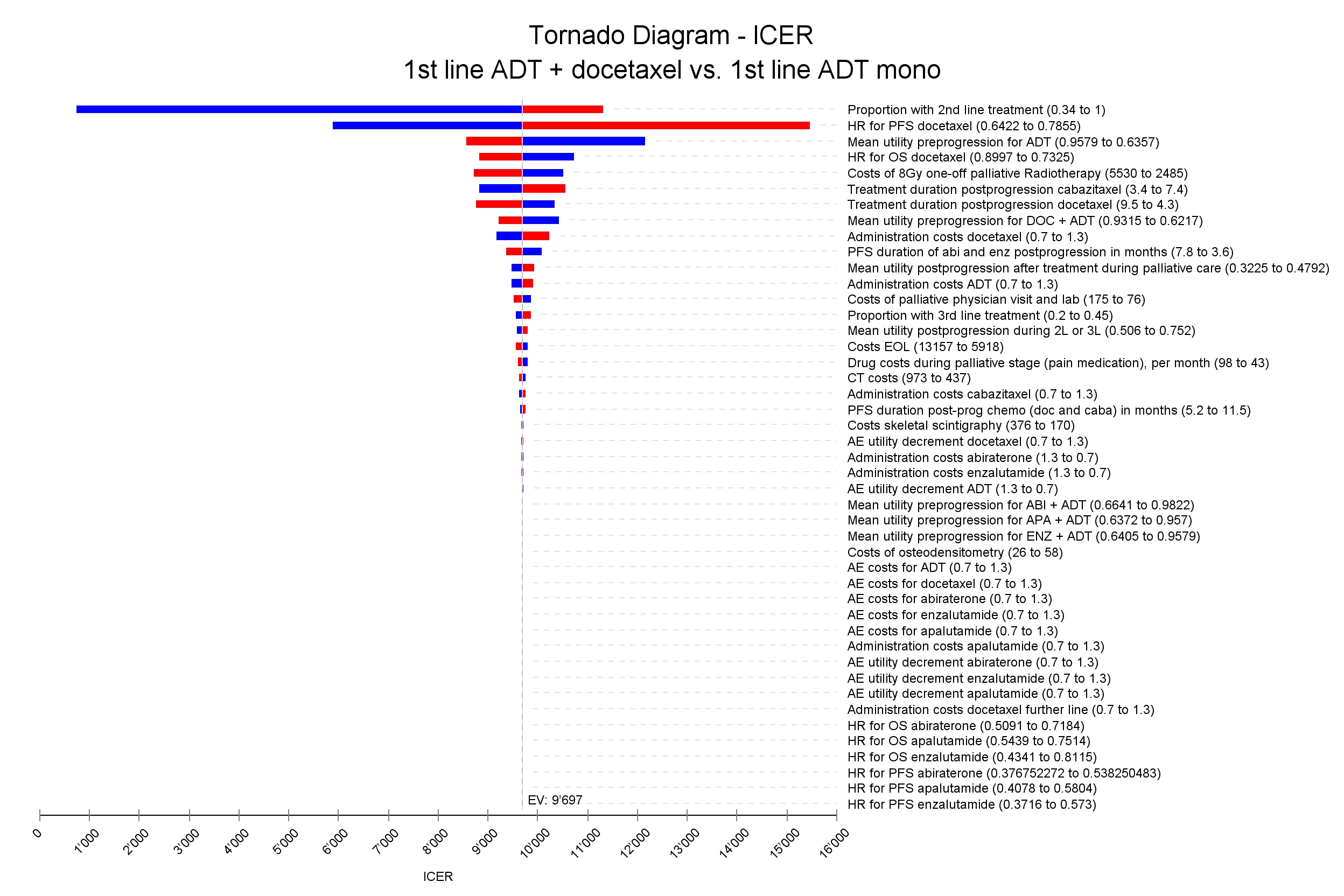


# S7 Fig. Tornado graph base case (ADT+docetaxel versus ADT).

ABI, abiraterone; ADT, androgen deprivation therapy; AE, adverse effect; APA, apalutamide; CT, computed tomography; ENZ enzalutamide; EOL end of life; EUR, euros; EV, expected value; HR, hazard ratio; ICER, incremental cost-effectiveness ratio; OS, overall survival; PFS, progression free survival; WTP, willingness-to-pay; 2L, second-line (corresponding to castration-resistant prostate cancer first-line).


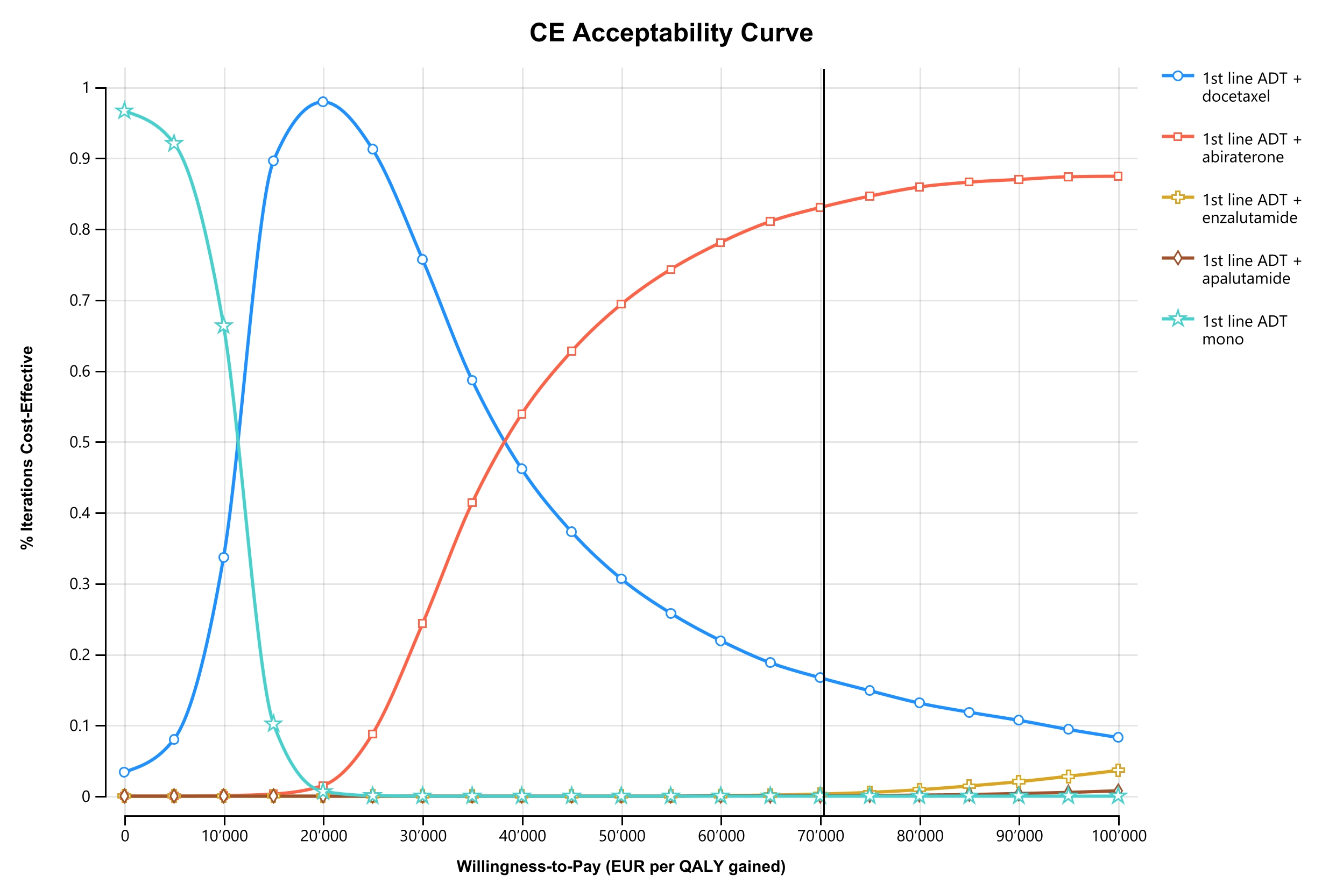


# S8 Fig. Cost-effectiveness acceptability curve

ADT, androgen deprivation therapy; EUR, euros; QALY, quality-adjusted life year.

# References

1. Clarke NW, Ali A, Ingleby FC, Hoyle A, Amos CL, Attard G, et al. Addition of docetaxel to hormonal therapy in low- and high-burden metastatic hormone sensitive prostate cancer: long-term survival results from the STAMPEDE trial. Ann Oncol. 2019. doi:10.1093/annonc/mdz396

2. James ND, de Bono JS, Spears MR, Clarke NW, Mason MD, Dearnaley DP, et al. Abiraterone for Prostate Cancer Not Previously Treated with Hormone Therapy. N Engl J Med. 2017;377: 338–351. doi:10.1056/NEJMoa1702900

3. Crawford ED, Schellhammer PF, McLeod DG, Moul JW, Higano CS, Shore N, et al. Androgen Receptor Targeted Treatments of Prostate Cancer: 35 Years of Progress with Antiandrogens. J Urol. 2018;200: 956–966. doi:10.1016/j.juro.2018.04.083

4. Vaishampayan UN, Heilbrun LK, Monk P, Tejwani S, Sonpavde G, Hwang C, et al. Clinical Efficacy of Enzalutamide vs Bicalutamide Combined With Androgen Deprivation Therapy in Men With Metastatic Hormone-Sensitive Prostate Cancer: A Randomized Clinical Trial. JAMA Netw open. 2021;4: e2034633. doi:10.1001/jamanetworkopen.2020.34633

5. Chi KN, Chowdhury S, Bjartell A, Chung BH, Pereira de Santana Gomes AJ, Given R, et al. Apalutamide in Patients With Metastatic Castration-Sensitive Prostate Cancer: Final Survival Analysis of the Randomized, Double-Blind, Phase III TITAN Study. J Clin Oncol. 2021; JCO.20.03488. doi:10.1200/jco.20.03488

6. Chi KN, Agarwal N, Bjartell A, Chung BH, Pereira de Santana Gomes AJ, Given R, et al. Apalutamide for Metastatic, Castration-Sensitive Prostate Cancer. N Engl J Med. 2019;381: 13–24. doi:10.1056/NEJMoa1903307

7. Menges D, Yebyo HG, Sivec-Muniz S, Haile SR, Barbier MC, Tomonaga Y, et al. Treatments for Metastatic Hormone-sensitive Prostate Cancer: Systematic Review, Network Meta-analysis, and Benefit-harm assessment. Eur Urol Oncol. 2022. doi:https://doi.org/10.1016/j.euo.2022.04.007

8. Wang L, Paller CJ, Hong H, De Felice A, Alexander GC, Brawley O. Comparison of Systemic Treatments for Metastatic Castration-Sensitive Prostate Cancer: A Systematic Review and Network Meta-analysis. JAMA Oncol. 2021;7: 412–420. doi:10.1001/jamaoncol.2020.6973

9. de Wit R, de Bono J, Sternberg CN, Fizazi K, Tombal B, Wülfing C, et al. Cabazitaxel versus Abiraterone or Enzalutamide in Metastatic Prostate Cancer. N Engl J Med. 2019;381: 2506–2518. doi:10.1056/nejmoa1911206

10. Akaza H, Procopio G, Pripatnanont C, Facchini G, Fava S, Wheatley D, et al. Metastatic castration-resistant prostate cancer previously treated with docetaxel-based chemotherapy: Treatment patterns from the PROXIMA prospective registry. J Glob Oncol. 2018;2018: 1–12. doi:10.1200/JGO.18.00009

11. Panje CM, Lupatsch JE, Barbier M, Pardo E, Lorez M, Dedes KJ, et al. A cost-effectiveness analysis of consolidation immunotherapy with durvalumab in stage III NSCLC responding to definitive radiochemotherapy in Switzerland. Ann Oncol Off J Eur Soc Med Oncol. 2020;31: 501–506. doi:10.1016/j.annonc.2020.01.007

12. Swiss Federal Statistical Office. CPI, Global index on all index bases. [cited 12 Jan 2022]. Available: https://www.bfs.admin.ch/bfs/de/home/statistiken/kataloge-datenbanken/tabellen.assetdetail.20944294.html

13. Organisation for Economic Co-operation and Development. OECD 6C Data. 2022. Available: https://data.oecd.org/conversion/purchasing-power-parities-ppp.htm

14. Woods BS, Sideris E, Sydes MR, Gannon MR, Parmar MKB, Alzouebi M, et al. Addition of Docetaxel to First-line Long-term Hormone Therapy in Prostate Cancer (STAMPEDE): Modelling to Estimate Long-term Survival, Quality-adjusted Survival, and Cost-effectiveness. Eur Urol Oncol. 2018;1: 449–458. doi:10.1016/j.euo.2018.06.004

15. Gravis G, Boher J-M, Joly F, Soulié M, Albiges L, Priou F, et al. Androgen Deprivation Therapy (ADT) Plus Docetaxel Versus ADT Alone in Metastatic Non castrate Prostate Cancer: Impact of Metastatic Burden and Long-term Survival Analysis of the Randomized Phase 3 GETUG-AFU15 Trial. Eur Urol. 2016;70: 256–262. doi:10.1016/j.eururo.2015.11.005

16. Gravis G, Fizazi K, Joly F, Oudard S, Priou F, Esterni B, et al. Re: Androgen-deprivation therapy alone or with docetaxel in non-castrate metastatic prostate cancer (GETUG-AFU 15): A randomised, open-label, phase 3 trial. J Urol. 2013;190: 2094. doi:10.1016/j.juro.2013.08.101

17. Kyriakopoulos CE, Chen Y-H, Carducci MA, Liu G, Jarrard DF, Hahn NM, et al. Chemohormonal Therapy in Metastatic Hormone-Sensitive Prostate Cancer: Long-Term Survival Analysis of the Randomized Phase III E3805 CHAARTED Trial. J Clin Oncol. 2018;36: 1080–1087. doi:10.1200/JCO.2017.75.3657

18. Sweeney CJ, Chen Y-H, Carducci M, Liu G, Jarrard DF, Eisenberger M, et al. Chemohormonal Therapy in Metastatic Hormone-Sensitive Prostate Cancer. N Engl J Med. 2015;373: 737–746. doi:10.1056/NEJMoa1503747

19. Fizazi K, Tran N, Fein L, Matsubara N, Rodriguez-Antolin A, Alekseev BY, et al. Abiraterone acetate plus prednisone in patients with newly diagnosed high-risk metastatic castration-sensitive prostate cancer (LATITUDE): final overall survival analysis of a randomised, double-blind, phase 3 trial. Lancet Oncol. 2019;20: 686–700. doi:10.1016/S1470-2045(19)30082-8

20. Fizazi K, Tran N, Fein L, Matsubara N, Rodriguez-Antolin A, Alekseev BY, et al. Abiraterone plus Prednisone in Metastatic, Castration-Sensitive Prostate Cancer. N Engl J Med. 2017;377: 352–360. doi:10.1056/NEJMoa1704174

21. James ND, de Bono JS, Spears MR, Clarke NW, Mason MD, Dearnaley DP, et al. Abiraterone for Prostate Cancer Not Previously Treated with Hormone Therapy. N Engl J Med. 2017;377: 338–351. doi:10.1056/NEJMoa1702900

22. Davis ID, Martin AJ, Stockler MR, Begbie S, Chi KN, Chowdhury S, et al. Enzalutamide with Standard First-Line Therapy in Metastatic Prostate Cancer. N Engl J Med. 2019;0: null. doi:10.1056/NEJMoa1903835

23. Armstrong AJ, Szmulewitz RZ, Petrylak DP, Holzbeierlein J, Villers A, Azad A, et al. ARCHES: A Randomized, Phase III Study of Androgen Deprivation Therapy With Enzalutamide or Placebo in Men With Metastatic Hormone-Sensitive Prostate Cancer. J Clin Oncol. 2019; JCO.19.00799. doi:10.1200/JCO.19.00799

24. Chi KN, Chowdhury S, Bjartell A, Chung BH, Pereira de Santana Gomes AJ, Given R, et al. Apalutamide in Patients With Metastatic Castration-Sensitive Prostate Cancer: Final Survival Analysis of the Randomized, Double-Blind, Phase III TITAN Study. J Clin Oncol. 2021; JCO.20.03488. doi:10.1200/jco.20.03488

25. Chi KN, Agarwal N, Bjartell A, Chung BH, Pereira de Santana Gomes AJ, Given R, et al. Re: Apalutamide for metastatic, castration-sensitive prostate cancer. J Urol. 2019;202: 661. doi:10.1056/NEJMoa1903307

26. Fizazi K, Scher HI, Molina A, Logothetis CJ, Chi KN, Jones RJ, et al. Abiraterone acetate for treatment of metastatic castration-resistant prostate cancer: final overall survival analysis of the COU-AA-301 randomised, double-blind, placebo-controlled phase 3 study. Lancet Oncol. 2012;13: 983–992. doi:10.1016/S1470-2045(12)70379-0

27. Collins R, Fenwick E, Trowman R, Perard R, Norman G, Light K, et al. A systematic review and economic model of the clinical effectiveness and cost-effectiveness of docetaxel in combination with prednisone or prednisolone for the treatment of hormone-refractory metastatic prostate cancer. Health Technol Assess (Rockv). 2007;11. doi:10.3310/hta11020

28. National Institute for Health Care Excellence (NICE). NICE Technology Appraisal: Enzalutamide for metastatic hormone-relapsed prostate cancer pre previously viously treated with a docetaxel [TA316]. 2014 [cited 1 Nov 2020]. Available: www.nice.org.uk/guidance/ta316

29. Swiss Federal Office of Public Health. Swiss Specialty List. [cited 11 Jan 2022]. Available: http://www.spezialitaetenliste.ch/ShowPreparations.aspx?searchType=SUBSTANCE

30. TARMED online Browser. [cited 11 Jan 2022]. Available: https://www.tarmed-browser.ch/de

31. Swiss Analysis List. [cited 11 Jan 2022]. Available: https://www.bag.admin.ch/bag/de/home/versicherungen/krankenversicherung/krankenversicherung-leistungen-tarife/Analysenliste.html

32. Barbier MC, Pardo E, Panje CM, Gautschi O, Lupatsch JE. A cost-effectiveness analysis of pembrolizumab with or without chemotherapy for the treatment of patients with metastatic, non-squamous non-small cell lung cancer and high PD-L1 expression in Switzerland. Eur J Heal Econ. 2021. doi:10.1007/s10198-021-01282-4

33. Sathianathen NJ, Alarid-Escudero F, Kuntz KM, Lawrentschuk N, Bolton DM, Murphy DG, et al. A Cost-effectiveness Analysis of Systemic Therapy for Metastatic Hormone-sensitive Prostate Cancer. Eur Urol Oncol. 2019. doi:10.1016/j.euo.2019.01.004

34. Sung WWY, Choi HCW, Luk PHY, So TH. A Cost-Effectiveness Analysis of Systemic Therapy for Metastatic Hormone-Sensitive Prostate Cancer. Front Oncol. 2021;11: 1–5. doi:10.3389/fonc.2021.627083

35. Chi KNN, Protheroe A, Rodríguez-Antolín A, Facchini G, Suttman H, Matsubara N, et al. Patient-reported outcomes following abiraterone acetate plus prednisone added to androgen deprivation therapy in patients with newly diagnosed metastatic castration-naive prostate cancer (LATITUDE): an international, randomised phase 3 trial. Lancet Oncol. 2018;19: 194–206. doi:10.1016/S1470-2045(17)30911-7

36. Morgans AK, Chen Y-H, Sweeney CJ, Jarrard DF, Plimack ER, Gartrell BA, et al. Quality of Life During Treatment With Chemohormonal Therapy: Analysis of E3805 Chemohormonal Androgen Ablation Randomized Trial in Prostate Cancer. J Clin Oncol. 2018;36: 1088–1095. doi:10.1200/JCO.2017.75.3335

37. Aguiar PN, Tan PS, Simko S, Barreto CMN, Gutierres B de S, Giglio A del, et al. Cost-effectiveness analysis of abiraterone, docetaxel or placebo plus androgen deprivation therapy for hormone-sensitive advanced prostate cancer. Einstein (São Paulo). 2019;17: eGS4414. doi:10.31744/einstein_journal/2019GS4414

38. Heijnsdijk EAM, Wever EM, Auvinen A, Hugosson J, Ciatto S, Nelen V, et al. Quality-of-Life Effects of Prostate-Specific Antigen Screening. N Engl J Med. 2012;367: 595–605. doi:10.1056/nejmoa1201637

39. Davies EW, Matza LS, Worth G, Feeny DH, Kostelec J, Soroka S, et al. Health state utilities associated with major clinical events in the context of secondary hyperparathyroidism and chronic kidney disease requiring dialysis. Health Qual Life Outcomes. 2015;13: 1–11. doi:10.1186/s12955-015-0266-9

40. Wehler E, Storm M, Kowal S, Campbell C, Boscoe A. Poster: A health state utility model estimating the impact of ivosidenib on quality of life in patients with relapsed/refractory acute myeloid leukemia. HemaSphere. 2018. p. 661. Available: https://library.ehaweb.org/eha/2018/stockholm/215730/michael.storm.a.health.state.utility.model.estimating.the.impact.of.ivosidenib.html#

41. Gravis G, Fizazi K, Joly F, Oudard S, Priou F, Esterni B, et al. Androgen-deprivation therapy alone or with docetaxel in non-castrate metastatic prostate cancer (GETUG-AFU 15): a randomised, open-label, phase 3 trial. Lancet Oncol. 2013;14: 149–158. doi:10.1016/S1470-2045(12)70560-0
