# Supplementary material for: Survival modelling and cost-effectiveness analysis of treatments for newly diagnosed metastatic hormone-sensitive prostate cancer
Source: PLoS One. 2022 Nov 3;17(11):e0277282. doi: 10.1371/journal.pone.0277282 (PMC9632884; doi:10.1371/journal.pone.0277282)
Supplement: Attachment — Submitted filename: Response to Reviewers_19.10.2022_final.docx [file pone.0277282.s003.docx]

PLOS ONE Reviewer comments on: “Survival modelling and cost-effectiveness analysis of treatments for newly diagnosed metastatic hormone-sensitive prostate cancer ”

No. PONE-D-22-24431

| **Comment number** | **Journal Requirements** | **The authors’ reply:** |
| --- | --- | --- |
| 1 | Please ensure that your manuscript meets PLOS ONE's style requirements, including those for file naming | We have updated the manuscript to meet PLOS ONE style requirements. |
| 2 | Please include captions for your Supporting Information files at the end of your manuscript, and update any in-text citations to match accordingly | We have included captions for Supporting Information files at the end of the document and have updated the in-text citations accordingly.  Since there are many supplementary text, table and figure files, we have grouped them into two main supplementary appendices for ease of access, and have additionally specified in the main document the individual text, figure or table number directly after the “hyperlinked” reference to the respective appendix (e.g. S2 Appendix S1 Text). If PLOS ONE prefers an individual referencing of each text/table/figure of the S1 Appendix, we are happy to comply with this, but would like to ask how to best deal, in this case, with the references only needed in individual supplementary text, table or figure files. |
| 3 | Please review your reference list to ensure that it is complete and correct. If you have cited papers that have been retracted, please include the rationale for doing so in the manuscript text, or remove these references and replace them with relevant current references. Any changes to the reference list should be mentioned in the rebuttal letter that accompanies your revised manuscript. If you need to cite a retracted article, indicate the article’s retracted status in the References list and also include a citation and full reference for the retraction notice | We checked the reference list of our manuscript and used the PLOS ONE reference style (from Mendeley). We deleted the one double reference as indicated in our answer to comment 4 of reviewer 1.  We have also corrected the URL link of reference 2 which was previously the same as for reference 3.  References 2 and 3 are internet pages whose contents (e.g. incidence numbers) have been slightly updated since this manuscript has been written. We have updated the numbers as follows on page 7 lines 121-124:  *In Switzerland, it has an age-standardized incidence of 118~~6~~.4~~1~~ per 100,000 person-years [2] and accounts for 28~~27.5~~% of all new male cancer cases (15~~14.9~~% across sexes) [3], placing a high burden on patients and the healthcare system. It also represents the second most frequent cause of death in men (15~~14.3~~%) [3].*  We have also updated the URL link of reference 22 (DRG Online Handbook) and of reference 38 (Wehler et al. Poster). |
|  |  |  |
|  | **Reviewer #1: Major Comments:** |  |
|  | Thank you for the opportunity to review the manuscript entitled, “Survival modelling and cost-effectiveness analysis of treatments for newly diagnosed metastatic hormone-sensitive prostate cancer”. Overall, this is an extremely thorough and well-structured model. The appendix is extensive and demonstrates the considerable thought and effort that was invested in this project. The BIA, while the numbers themselves not being specifically relevant to the majority of readers, highlights the overall impact of these agents on funding decisions within a healthcare budget. There are a few questions that this paper raised which are outlined below: | Thank you very much for this comment. |
| 1 | What do you mean by re-creating individual patient OS and PFS data from RCTs? Simply clarify if access to individual patient level data was obtained because this component of the methods is slightly unclear. | We did not have access to individual patient level data.  We digitalised published OS and PFS curves from the corresponding manuscripts with a software called DigitizeIt with which we obtained x and y values of each curve. We then applied the statistical algorithm of Guyot et al. (<https://doi.org/10.1186/1471-2288-12-9>), implemented as an R program, to the digitised data to back-develop the Kaplan Meier curves (using where available information on number of events and numbers at risk). The Guyot procedure is of course not able to perfectly re-create the original patient level data, but is able to approximate them and then construct OS and PFS curves which are e.g. in their median, at the different time points and also overall similar to the original curves. This is a standard approach applied when individual data is not available (e.g. NICE Single Technology Appraisal ID1609 Nivolumab with ipilimumab for untreated unresectable malignant pleural mesothelioma <https://www.nice.org.uk/guidance/ta818/evidence/appraisal-consultation-committee-papers-pdf-11188250029>).  We added in the manuscript (version with visible track changes p.10, l.193) that we did not have access to individual patient data to make this point more clear:  *We did not have access to individual patient data and therefore digitalized published Kaplan-Meier (KM) OS and PFS curves (Software DigitizeIt [25]) to re-create IPD (method of Guyot et al. [26]).* |
| 2 | What is the rationale for 1 month cycle lengths? | Since the OS and PFS curves used for re-creation (of OS and PFS data) were published on a monthly basis (on the x-axis), we could (re-)create monthly OS and PFS data without a need for conversion. We calculated all costs and QALYs per month and then added them up as appropriate. Overall, the monthly cycle length allowed for a certain increase in precision and had no disadvantages, apart from a marginal increase in computation time that was only of practical relevance for the probabilistic sensitivity analysis.  Changes performed within the manuscript: None |
| 3 | Further elaboration and presentation of the supporting evidence for the choice of a higher utility for the ADT+abiraterone strategy would be worthwhile as the conclusion does change with variation of this one variable | Sathianathen et al. 2019 (Indirect Comparisons of Efficacy between Combination Approaches in Metastatic Hormone-sensitive Prostate Cancer: A Systematic Review and Network Meta-analysis; doi:10.1016/j.eururo.2019.09.004) stated in the discussion of their article that *“...the Latitude trial (Chi et al. 2018, Patient reported outcomes following abi+prednisolone;* [*http://www.ncbi.nlm.nih.gov/pubmed/31150574*](http://www.ncbi.nlm.nih.gov/pubmed/31150574)*) reported that patients on abiraterone acetate had a consistently better quality of life compared with those receiving ADT only at most time points measured. Meanwhile, apalutamide was shown to be comparable to ADT only from a quality-of-life perspective [based on Chi et al. 2019, Apalutamide for Metastatic, Castration-Sensitive Prostate Cancer].”*  On page 13 line 259 (utility chapter) we have now added the above mentioned Sathianathen 2019 and Chi 2018 and 2019 publications as references too.  In S10 Table (page 16 S2 Appendix) we also added the Chi 2019 reference as a justification for using the same utility for ADT+apalutamide as for ADT. |
| 4 | Copy Editing Comments: • Reference 32 and 34 are the same paper • Line 356-357 is missing a word | - Thank you very much for this comment. We took the double reference out. - *We have re-formulated the sentence p. 20 l. 395 from:*   *“Compared with ADT monotherapy only and from a Canadian perspective, ADT+apalutamide treatment showed not cost-effective versus ADT [47].”*  To:  *“Compared with ADT monotherapy only and from a Canadian perspective, ADT+apalutamide treatment* ~~showed not~~ was unlikely *to be cost-effective versus ADT [47].”* |
|  |  |  |
|  | **Reviewer #2 Major Comments:** |  |
| 1 | Overall, the authors have performed a detailed CEA analysis and the methodology appears to be robust. The most important clinical (1L, 2L, palliative, AE) and methodological considerations appear to be well captured. | Thank you very much for this feedback. |
| 2 | Novelty: The largest criticism is relative to the impact of the manuscript. While the methodology itself appears well done, as the authors note in their Discussion, multiple prior CEAs on this topic have been previously reported. In addition, the prior CEAs reach overall similar conclusions to the one presented here, though the authors are able to report increased follow-up and better refine the inputs. Furthermore, with the similar estimated survival curves (Fig 2), it is not surprising that abiraterone is shown to be the most cost-effective ARAT strategy given the cost benefit and the modelled utility benefit. | Thank you for this comment. We agree that several CEAs on this topic have been performed in different countries, with similar results. Nevertheless, we believe it is important to publish our results for several reasons. As stated in the discussion of our manuscript:  “Our approach is innovative in that it evaluated all currently available treatments for mHSPC simultaneously in a European setting including more mature clinical data compared to earlier analyses and the new abiraterone generic price in the base case. In addition, our model investigated the impact of the new abiraterone price on the possibility of apalutamide and enzalutamide to become a cost-effective or even the preferred treatment options. Further strengths are a refined survival analysis (meta-analysis of recreated and most up-to-date individual survival data of all current mHSPC treatments, comparison of different survival models and curve extrapolations), a homogenous PFS definition (cPFS, rPFS) and that the model parameters were based on a full-scale, systematic health economic literature review.”  We all know that research is a continuous process, with the availability of more information (e.g., longer follow-up, updated costs) allowing for more accurate calculations/predictions. The fact that the results remain similar confirms the results of previous work, which were based on less information (e.g., shorter follow-up) and more assumptions.  In addition, we think that it is worth noting that our model was carefully developed in the Swiss context (especially in terms of costs input). While not many countries may be very comparable in terms of healthcare organization and expenditures, the results may still be an important reference point for different European countries.  Changes performed within the manuscript: None |
| 3 | The other most significant limitation is the duration of the extrapolation relative to the modelling horizon. This is touched upon in the Discussion for enzalutamide primarily, but remains an important challenge for all arms. | We fully agree with this comment. The stated limitation applies to all CEAs trying to estimate long-term treatment effects based on data with limited observation times. However, for treatment decisions with a long-term impact on survival and/or QoL, the only appropriate time horizon is life-long; everything else might lead to biased results. The uncertainty arising from the extrapolation needs to be clearly recognised as a substantial limitation (outlined in the discussion section), but is unavoidable.  In our case, this limitation was particularly true for enzalutamide and slightly less for apalutamide because the median follow-up times were relatively short (ADT+enzalutamide: median follow-up time of 14.4 months; ADT+apalutamide: 22.7 months for PFS). For the other strategies considerably longer periods of observation were available. For these, the uncertainty in the extrapolation should be more limited.  Changes performed within the manuscript:  On p. 21 l. 413-414 we added the following text in red:  *Any results and conclusions drawn from our model for enzalutamide treatment, but also for the other treatments, thus need to be treated with caution, until longer-term data is available.* |
| 4 | (Minor) More details regarding AE should be included – how they were modelled – incidence, could patients have multiple, etc.? They are only briefly covered in the A5 Supplement. | Thank you for this comment.  In order to make the AE derivation more clear, we updated the text in the S5 Text and also made a slight adaptation in the discussion section of the main manuscript. The monthly incidence rates for ADT and the incidence rate ratios for the combination treatments are presented in the S2 Table.  In the main manuscript, p.21 l.422-423 (discussion section), we made the following adaptation:  This may have led to a certain degree of bias~~overestimation~~ present in the modelled AE rates.  We suggest to change the S5 text from:  **Supplementary Section A5 Adverse effects**  In the health economic model, we only considered adverse effects (AEs) until disease progression and not for treatment lines thereafter. The use of AE grades 3-5 instead of grades 3-4, necessitated by the approach to reporting in some trial publications, implied overestimation. However, fatal (grade 5) AEs were infrequent in the trials, so that we did not expect them to have strongly influenced the overall AE rates for the described outcomes.  Monthly AE incidence rates were again derived from our meta-analysis of AEs by Menges et al. [7]. The analysis used the number of events in each group divided by the person-time at risk (i.e., the total of number participants in a group multiplied by the median follow-up time). Again, the LATITUDE, ENZAMET, and NCT02058706 trials were excluded.  To:  **S5 Text. Adverse effects.**  For the health economic model, we selected adverse effects (AEs) of severity grades 3-5, which we considered important based on the opinions of involved Swiss medical experts and given their potential of leading to hospitalizations. The use of AE grades 3-5 (including AE-related deaths), instead of grades 3-4, was necessitated by the limited reporting in some trial publications and may have led to an overestimation of AE rates. However, fatal (grade 5) AEs were infrequent in the trials, so that we do not expect them to have strongly influenced the overall AE rates for the described outcomes. We only considered AEs until disease progression, but not for treatment lines thereafter.  Monthly AE incidence rates for each adverse event type were derived from our meta-analysis of AEs [7] in the form of monthly incidence rates for patients receiving ADT alone and treatment-specific rate ratios for the other treatment strategies (S2 Table). Incidence rates per trial arm and per AE type were calculated as the number of persons with AE events for each treatment group divided by the person-time at risk (i.e., the total number of participants per treatment group multiplied by the median follow-up time). From these estimates, we then estimated incidence rates for the ADT group across all trials as well as incidence rate ratios for each of the combination treatments by calculating a weighted mean estimate.  The AE calculation was limited by the reporting in some of the relevant trials (i.e., for each individual AE type, trials typically reported the AE of highest severity if multiple AEs of the same type occurred in a patient). In consequence, the resulting estimates of monthly incidence rates may be false low.  Again, the LATITUDE, ENZAMET, and NCT02058706 trials were excluded, while the CHAARTED, GETUG, STAMPEDE, ARCHES, and TITAN trials were included (depending on the coverage of the specific AE type in the publications). |
| 5 | (Minor) Can the authors provide the tunnels/formulae construction for 1L vs 2L vs palliative for CRPC given that they were not separate health states. | To model post-progression treatments (CRPC 1L and CRPC 2L) and palliative care we applied the assumptions outlined in S3 to S5 Tables.  Only a certain percentage of the patients who entered the PD state received one or several 2L (or higher) treatments, all others received palliative treatment directly (S4 Table). For the patients who received post-progression treatments, the treatments as specified in S3 Table were assumed. Since there were no separate health states assumed, we modelled the durations of further line treatments as outlined in S5 Table.  On page 9 line 165 we made the following adaptions in red:  The additional lines of treatment for CRPC were not represented in the model by separate health states but covered ‘within’ the PD state using tunnel states and appropriate formulae (section treatment strategies).  In S2 Appendix we added the following text at the end of S6 Text:  All derived inputs for further line treatments were then entered into the TreeAge model which used tunnel states to remember for all sub-fractions of the modelled cohorts when they have entered a given health state. On this basis, treatment costs and utilities can be modelled dependent on the time already spent in the respective health state. |
